# Identification of Bioactive Compounds from the Roots of *Rehmannia glutinosa* and Their In Silico and In Vitro AMPK Activation Potential

**DOI:** 10.3390/molecules29246009

**Published:** 2024-12-20

**Authors:** Hwaryeong Lee, Isoo Youn, Sang Gyun Noh, Hyun Woo Kim, Eunhye Song, Sang-Jip Nam, Hae Young Chung, Eun Kyoung Seo

**Affiliations:** 1 Graduate School of Pharmaceutical Sciences, College of Pharmacy, Ewha Womans University, Seoul 03760, Republic of Korea; jongsky119@naver.com (H.L.); iyoun@ewha.ac.kr (I.Y.); seh0413@ewhain.net (E.S.); 2 Department of Pharmacy, College of Pharmacy, Pusan National University, Busan 46241, Republic of Korea; rskrsk92@pusan.ac.kr (S.G.N.); khw124124@naver.com (H.W.K.); hyjung@pusan.ac.kr (H.Y.C.); 3 Department of Chemistry and Nanoscience, Ewha Womans University, Seoul 03760, Republic of Korea; sjnam@ewha.ac.kr

**Keywords:** *Rehmannia glutinosa*, uridine derivative, iridoid glycoside, phenylpropanoid glycosides, AMP-activated protein kinase

## Abstract

*Rehmannia glutinosa* Libosch., which belongs to the Orobanchaceae family, is a perennial herb found in China, Japan, and Korea. In traditional medicine, it is used to cool the body, improve water metabolism in the kidney, and provide protection from metabolic diseases such as type 2 diabetes mellitus (T2DM) and obesity. In this study, three new compounds were isolated from the roots of *R. glutinosa*, along with eighteen known compounds. Structure elucidation was performed with spectroscopic analyses including nuclear magnetic resonance (NMR) and circular dichroism (CD) spectroscopy. As the AMP-activated protein kinase (AMPK) signaling pathway is reportedly related to metabolic diseases, AMPK activation studies were conducted using in silico simulations and in vitro assays. Among the isolated compounds, **1** showed a potential as an AMPK activator in both in silico simulations and in vitro experiments. Our findings expand the chemical profiles of the plant *R. glutinosa* and suggest that one newly found compound (**1**) activates AMPK.

## 1. Introduction

*Rehmannia glutinosa* Libosch. (Orobanchaceae) is a perennial flowering plant, and its distribution is wide spread in China, Japan, and Korea [1]. The chemical classes of the compounds typically found in this plant are iridoids, ionones, phenylethanoids, and phenylpropanoids [2]. *R. glutinosa* is used in traditional medicine to treat excessive heat from the body and unbalanced water metabolism in the kidney [3]. In modern medicine, it has been reported to have hypoglycemic, anti-inflammatory, angiogenetic, and hepatoprotective effects [1]. As anti-diabetic and anti-obese effects of *R. glutinosa* have drawn attention among researchers [4,5,6,7,8], finding biologically active ingredients and investigating the mechanisms of action by compounds have become fascinating areas of interest.

Metabolic diseases are characterized by unusual metabolism, which means the overwhelming chemical reactions within cells during digestion and energy homeostasis [9]. Obesity, T2DM, hyperlipidemia, cancers, and cardiovascular diseases (CVD) are considered metabolic diseases, and Kazibwe et al. have revealed that the average costs/year of CVD, cancers, and T2DM patients were USD 6056, USD 3304, and USD 1017, respectively [10]. Therefore, the prevention and proper treatment of these diseases are primary global concerns. Natural products can be a source of drug discovery for metabolic diseases by offering diverse drug scaffolds with mild side effects [11]. For example, *Costus igneus*, which is called “insulin plant” in India, has been used as an herbal cure for T2DM [12], and Youn et al. showed anti-adipogenic and thermogenic activities in the 3T3-L1 cells of the extract and compounds from *Perilla frutescens* var. *acuta* [13].

AMP-activated protein kinase (AMPK) is a cellular energy sensor and activated by energy-lowering changes in the body, such as malnutrition and hypoxia [14]. AMPK plays a critical role in maintaining energy homeostasis during metabolic stress. As the functional aspects of AMPK have been revealed to be related to glucose/lipid homeostasis, body weight, food intake, insulin signaling, and mitochondrial biogenesis, AMPK has become a major therapeutic target for the treatment of metabolic diseases, including T2DM and obesity [15,16].

In silico simulations help to predict the potential of a constituent as a drug by calculating the interaction of a ligand with its receptor, and the absorption, distribution, metabolism, excretion, and toxicity (ADMET) of a drug [17]. Molecular dynamics can also be applied through in silico docking to calculate the behavior of molecules over time by predicting system energy and behavior. These approaches can predict a drug target, such as receptors and enzymes, and behavior of a drug in the body. Appropriate in vitro or in vivo investigations should be requested to support the results from in silico simulations.

**Figure 1:**
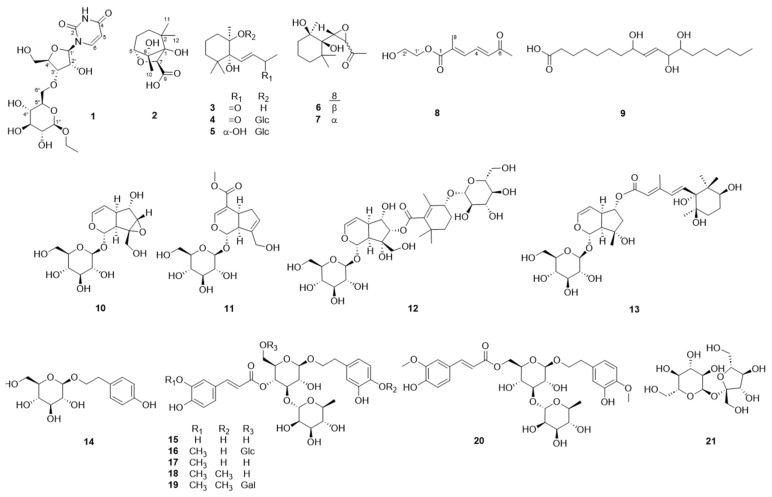
Structures of the isolated compounds **1**–**21**.

In this study, compounds in *R. glutinosa* were isolated to clearly identify its active ingredients (Figure 1). Structures were identified using 1D/2D NMR and high-resolution electrospray ionization mass spectrometry (HR-ESIMS). The binding energies of the isolated compounds and AMPK receptor were calculated using in silico docking simulations. Moreover, a full set of molecular dynamics and ADMET predictions were performed on compound **1** to predict its AMPK-activating effect. The results from the simulation were also explored by an in vitro AMPK activation assay.

## 2. Results

### 2.1. Structure Elucidation

**Figure 2:**
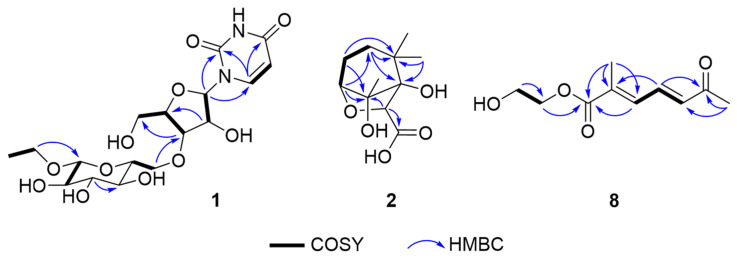
COSY and HMBC key correlations of compounds **1**, **2**, and **8**.

**Figure 3:**
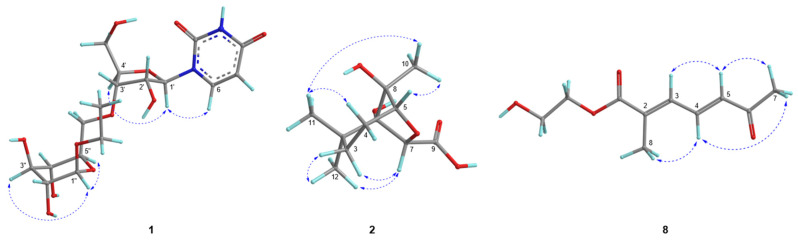
Nuclear Overhauser effect correlations of compounds **1**, **2**, and **8**.

Compound **1** was obtained as a brown gum. HR-ESIMS [M + 2H_3_O]^2+^ at *m*/*z* 236.1232 and ^13^C NMR data suggested a molecular formula of C_17_H_26_N_2_O_11_. Compound **1** showed characteristic signals for uridine in the ^1^H- and ^13^C-NMR spectra as follows: *δ*_C_ 152.5 (C-2); 166.3 (C-4); *δ*_H_ 5.69 (1H, d, *J* = 8.0 Hz)/102.7 (CH-5); 8.00 (1H, d, *J* = 8.0 Hz)/142.8 (CH-6); 5.90 (1H, d, *J*_1′,2′_ = 4.7 Hz)/90.8 (CH-1′); 4.17 (1H, ddd, *J*_1′,2′_ = 4.7 Hz, *J*_2′,3′_ = 4.6 Hz)/75.8 (CH-2′); 4.15 (1H, ddd, *J*_2′,3′_ = 4.6 Hz, *J*_3′,4′_ = 4.5 Hz)/71.4 (CH-3′); 4.00 (1H, dt, *J* = 4.5, 3.0 Hz)/86.4 (CH-4′); 3.83 (1H, dd, *J* = 12.2, 3.0 Hz); and 3.73 (1H, dd, *J* = 12.2, 3.0 Hz)/62.3 (CH_2_-5′) [18,19]. H-2′ and H-3′ included peaks that skewed toward each other in the ^1^H-NMR spectrum, and their *J* value, along with the dihedral angles (−33.1°) predicted by Chem 3D, also supported a moiety associated with uridine. The presence of β-glucopyranoside was supported by ^1^H- and ^13^C-NMR data, and the attachment of this moiety was determined to be the 3′-OH position of uridine ribofuranose by the HMBC correlation between H-6″a (*δ*_H_ 3.66 ppm) and C-3′ (*δ*_C_ 71.4 ppm) (Figure 2) [20]. The three-bond correlation between CH_3_CH_2_O-1” and C-1” in the HMBC spectrum supported the assignment of the ethyl group in the structure to be an anomeric hydroxyl group of glucopyranoside (Figure 2). In the NOESY spectrum, the correlation between H-1′ and H-4′ supported the aglycone group as uridine, and the cross-peaks between H-1″, H-3″, and H-5″ suggested the presence of glucopyranoside (Figure 3). The circular dichroism (CD) spectrum of compound 1 exhibited a positive Cotton effect at 273 nm and a negative Cotton effect at 220 and 243 nm, which was similar to the CD spectroscopy trend of uridine [21]. Compound **1** was therefore determined to be 1-*O*-ethyl-β-d-glucopyranosyl-(6 → 3′)-uridine (Figure 1).

The molecular formula of compound **2** was determined to be C_11_H_18_O_5_ based on the HR-ESIMS [M + Na]^+^ ion at *m*/*z* 253.1051. The ^1^H-NMR spectrum of compound **2** showed three methyls [*δ*_H_ 1.52 (3H, s, H-10), 1.05 (3H, s, H-12), and 1.04 (3H, s, H-11)], two methylenes [*δ*_H_ 1.93 (1H, m, H-3b), 1.90 (1H, m, H-4b), 1.82 (1H, m, H-4a), and 1.37 (1H, dddd, *J* = 11.4, 6.7, 2.1 Hz, H-3a)], and two methines [*δ*_H_ 4.20 (1H, s, H-7) and 4.06 (1H, d, *J* = 5.6 Hz, H-5)]. A ^13^C-NMR spectrum revealed signals of eleven carbons, including a carbonyl carbon at *δ*_C_ 174.8 (C-9) and four oxygenated carbons, two quaternary carbons at *δ*_C_ 92.1 (C-8) and 85.8 (C-1), and two methine carbons at *δ*_C_ 81.9 (C-5) and 81.4 (C-7). Key correlations of COSY were shown between H-3a/H-4a and H-4b/H-5 (Figure 2). In addition, the correlations of H-3a/C-1, H-4b/C-2, and C-8; H-5/C-1, C-3, C-7, and C-8; and H-7/C-8 in the HMBC spectrum provided crucial information to elucidate the structure of compound **2** as an *O*-bridged bicyclic skeleton (Figure 2). The HMBC correlation from H-7 to C-9 was indicative of the attachment of a carbonyl group at C-7. Correlations of H-11/C-12, H-12/C-1, C-2, C-3, and C-11, and H-10/C-1, C-5, and C-8 in the HMBC spectrum suggested the attachment of three methyl groups at C-2 and C-8. The key nuclear Overhauser effect (NOE) correlations between H-7/H-3b and H-12, H-4a/H-10 and H-11, H-5/H-10, and H-10/H-11 helped to assign the relative configuration of compound **2**, confirming H-3a, H-4a, H-5, H-10, and H-11 oriented in the same direction (Figure 3). In a further study, the energy-minimization of compound **2,** which was determined using Chem3D 21.0.0 software, corresponded with those of the experimental results. Compound **2** was named 1,8-dihydroxy-2,2,8-trimethyl-6-oxabicyclo [3.2.1]octane-7-carboxylic acid.

A molecular formula of C_10_H_14_O_4_ was established for compound **8** based on the molecular ion peak at *m*/*z* 100.0519 [M + 2H]^2+^ derived from HR-ESIMS. The ^1^H- and ^13^C-NMR signals exhibited two methyls [*δ*_H_ 2.33/*δ*_C_ 27.6 (CH_3_-7) and 2.11/13.8 (CH_3_-8)], two methylenes [*δ*_H_ 3.39/*δ*_C_ 43.5 (CH_2_-1′) and 3.64/61.5 (CH_2_-2′)], and three olefinic groups [*δ*_H_ 7.56/*δ*_C_ 139.1 (H-4), 6.92/131.7 (H-3), and 6.38/135.1 (H-5)]. This suggested that three protons (H-3, 4, and 5) are connected in a *trans*-form diene based on the *J* values of the three olefinic protons (*J*_3,4_ = 11.5 Hz and *J*_4,5_ = 15.6 Hz) and NOESY correlations between H-3/H-5 (Figure 2 and Figure 3). In the HMBC spectrum, the attachments of carbonyl carbons at C-2 and C-5 were supported by the correlations from H-4, H-5, and H-7 to C-6 and from H-3 and H-8 to C-1, respectively (Figure 2). The position of an ethyl moiety was assigned by the HMBC correlations between H-1′ and C-1, and the downfield-shifted chemical shift value of C-2′ proved the existence of a hydroxyl group at C-2′. This was also supported by HR-ESIMS fragmentation traces. A thorough analysis of the 1D and 2D NMR spectra of compound **8** revealed it to be 2′-hydroxyethyl (2*E*,4*E*)-2-methyl-6-oxohepta-2,4-dienoate, a structure similar to organic compounds identified in the reaction mechanisms and synthetic pathways of the previous study (Figure 1) [22].

Along with three newly identified compounds, 18 previously known compounds were isolated (Figure 1): dihydroxy-β-ionone (**3**) [23], rehmaionoside C (**4**) [23], rehmaionoside A (**5**) [24], frehmaglutin J (**6**) [25], frehmaglutin I (**7**) [25], tianshic acid (**9**) [26], catalpol (**10**) [27], geniposide (**11**) [28], frehmaglutoside G (**12**) [29], 6-*O*-sec-hydroxyaeginetoyl ajugol (**13**) [30], salidroside (**14**) [20], acteoside (**15**) [31], jionoside A_1_ (**16**) [32], leucosceptoside A (**17**) [33], martynoside (**18**) [34], jionoside B_1_ (**19**) [32], isomartynoside (**20**) [35], and sucrose (**21**) [36]. Compound **9** was found for the first time in the *R. glutinosa* species.

### 2.2. In Silico Molecular Docking, MD, and ADMET Simulations

**Figure 4:**
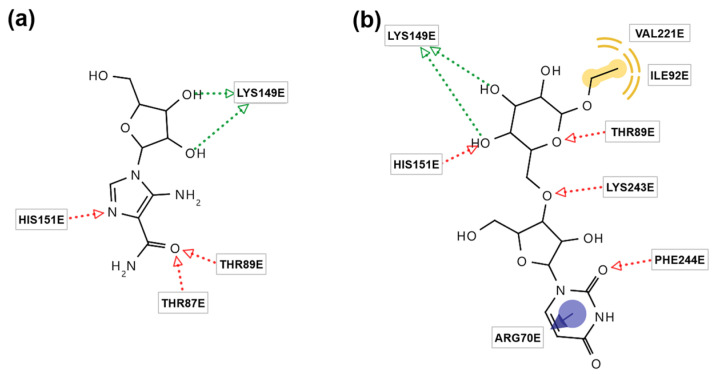
Binding interactions of (**a**) 5-amino-4-imidazolecarboxamide ribonucleoside (AICAR) and (**b**) compound **1** with AMP-activated protein kinase (AMPK) in silico docking simulations in Autodock 4.2. Green arrow: hydrogen bond (H-bond) donor; red arrow: H-bond acceptor; yellow interaction: hydrophobic interaction or van der Waals force; blue arrow: aromatic interaction. (ARG, arginine; HIS, histidine; ILE, isoleucine; LYS, lysine; PHE, phenylalanine; THR, threonine; VAL, valine).

As various approaches to in silico docking simulations were explored, the binding energies of compounds **1**–**21** were predicted against AMPK by three software programs (Autodock Vina 1.1.2, Autodock 4.2, and Dock 6.12). Among the compounds, **1** (−11.4 Kcal/mol) showed stronger affinities with AMPK than those of the control (5-amino-4-imidazolecarboxamide ribonucleoside, AICAR), as shown in Table 1. Compound **1** formed a higher number of hydrogen bonds with AMPK than the control, indicating more stable interactions of compound **1** with AMPK. It also exhibited hydrophobic/van der Waals and aromatic interactions with AMPK. In other words, compound **1** has the potential for strong activity against AMPK in the molecular interaction models (Figure 4). As both AICAR and compound **1** formed the hydrogen bonds with LYS149 in AMPK, LYS149 strengthens the interaction with a ligand and helps AICAR and compound **1** to serve as an AMPK activator.

**Figure 5:**
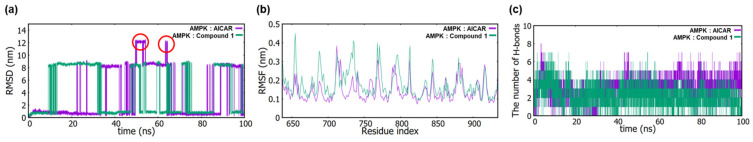
Molecular dynamics study of AICAR and compound **1** against the AMPK receptor. (**a**) Root-mean-square deviation (RMSD) plots of the target protein (AMPK) and ligands (AICAR or **1**) complex over 100 ns MD simulation. A red circle indicates an unstable region. (**b**) Root-mean-square fluctuation (RMSF) plots of AMPK and the ligands (AICAR and **1**). (**c**) Number of hydrogen bonds of the ligands (AICAR and **1**) with AMPK.

In molecular dynamics, the root-mean-square deviation (RMSD) is an indicator of the stability of a protein complex. The smaller the RMSD value is, the more stable the interaction becomes [37]. Although AICAR and compound **1** usually showed different stability during the simulation (100 ns), unstable regions were observed around 50 and 60 ns for AICAR, which means compound **1** forms a more stable complex with the AMPK protein. Root-mean-square fluctuation (RMSF) quantifies how much a ligand moves from its average position over time in a molecular dynamics simulation [38]. As compound **1** shared similar fluctuation patterns in the several residue index, it can have a similar activity as the AICAR against AMPK (Figure 5b). The number of hydrogen bonds between the ligands (AICAR and compound **1**) and AMPK were predicted. As shown in Figure 5c, compound **1** usually formed more hydrogen bonds with AMPK in 0–30 ns, and AICAR established more hydrogen bonds with AMPK after 40 ns. These results indicated that both AICAR and compound **1** can be potent AMPK activators.

In silico physicochemical and ADMET profiling of compound **1** is shown in Table S1. The ADMET results support the drug-likeness of a drug candidate by describing how it interacts with the body over time. Compound **1** showed good absorption, distribution, metabolism, excretion, and toxicity in general.

### 2.3. In Vitro AMPK Activation Assay

**Figure 6:**
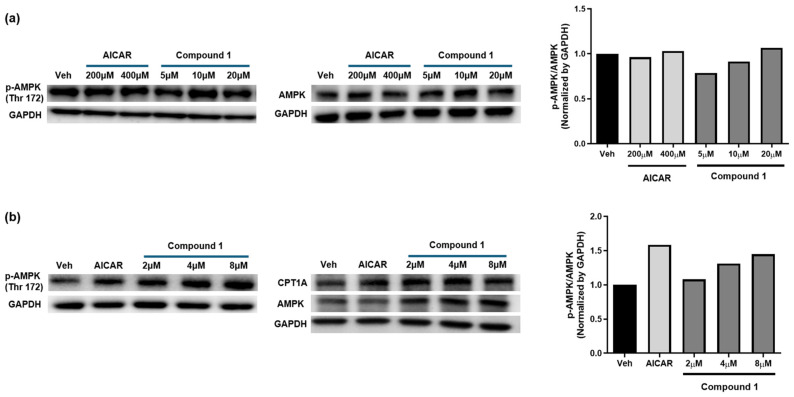
AMPK activation by AICAR (the control) and compound **1**. The AMPK activation was evaluated by measuring AMPK phosphorylation (pAMPK) in the presence of AICAR and compound **1** using Western blotting. (**a**) The concentration of AICAR was 200 and 400 μM. The concentrations of compound **1** were 5, 10, and 20 μM. The ratio of p-AMPK/AMPK was measured after normalization of p-AMPK and AMPK to GAPDH, respectively. (**b**) The concentration of AICAR was 500 μM, and the concentrations of compound **1** were 2, 4, and 8 μM. p-AMPK: phosphorylated AMPK. CPT1A: carnitine palmitoyl transferase 1A.

HepG2 cells were treated with the serial concentrations of compound **1** (5, 10, and 20 μM) to investigate its effects against AMPK. The AMPK activation was evaluated by measuring AMPK phosphorylation (pAMPK) in the presence of AICAR (the control) and compound **1** using Western blotting. The concentration of AICAR was 200 and 400 μM, and the concentrations of compound **1** were 5, 10, and 20 μM. Compound **1** increased the phosphorylation of AMPK, and thus, the p-AMPK/AMPK ratio increased in a dose-dependent manner (Figure 6a). In the next round, the concentration of AICAR was 500 μM, and the concentrations of compound **1** were 2, 4, and 8 μM. AMPK phosphorylation was stimulated by compound **1**, and the ratio of p-AMPK/AMPK also grew dose-dependently, as shown in Figure 6b. Moreover, the protein level of carnitine palmitoyl transferase 1A (CPT1A) was increased because AMPK is tightly involved in regulating CPTA1.

## 3. Discussion

In this study, three new compounds were isolated from *R. glutinosa*: 1-*O*-ethyl-β-D-glucopyranosyl-(6 → 3′)-uridine (compound **1**), 1,8-dihydroxy-2,2,8-trimethyl-6-oxabicyclo[3.2.1]octane-7-carboxylic acid (compound **2**), and 2′-hydroxyethyl-(2*E*,4*E*)-2-methyl-6-oxohepta-2,4-dienoate (compound **8**). Both compounds **1,** a glycosylated uridine, and compound **2,** an *O*-bridged bicyclo-octane, are rare in nature. Iridoid glycosides (compounds **10**–**13**) and phenylpropanoid glycosides (**14**–**20**), which are found widely in this species, were also isolated in this study. Among the known compounds, compound **9** was isolated for the first time in this study. Catalpol (compound **10**) reportedly exhibits anti-inflammatory and antioxidant activities [39], and geniposide (**11**) has shown protective effects on cerebral diseases [40]. Moreover, salidroside (compound **14**) has shown beneficial effects against ischemic diseases [41], and acteoside (compound **15**) has been reported to have cardioprotective, anti-diabetic, and neuroprotective effects [42].

To the best of our knowledge, this is the first report of in silico docking simulations of compounds **1**–**21** against AMPK as a receptor. Among the ingredients in *R. glutinosa*, catalpol (compound **10**) has been studied using in silico docking simulations as a ligand against the glutathione peroxidase receptor due to its neuroprotective effect [43], and the involvement of rhein in the absorption of rehmannioside D was investigated using in silico approaches [44]. This study provides a full set of in silico molecular-docking simulations, along with the binding energies between the isolated compounds and AMPK, the binding interactions of compound **1** and AMPK, the molecular dynamics of compound **1**, and the ADMET of compound **1**. In molecular dynamics, compound **1** was suggested as an effective AMPK activator because its RMSD and RMSF values and its number of hydrogen bonds were similar or higher than those of AICAR, a widely known AMPK activator.

The in vitro assay was performed to confirm the AMPK activation of compound **1**, as predicted in the in silico docking simulation results. As expected, compound **1** increased the AMPK phosphorylation at 5, 10, and 20 μM. It also stimulated the AMPK phosphorylation at 2, 4, and 8 μM. In a previous study, catalpol (**10**) decreased blood glucose levels and mitigated insulin resistance by recruiting the AMPK signaling pathway [45,46]. A more detailed activity study of compound **1** will therefore be needed to meet the goal as a treatment for metabolic diseases by identifying the mechanisms of action and structure-activity relationships, along with in vivo AMPK-activating effects.

Although *R. glutinosa* has been widely investigated, its chemical library can be expanded by isolating structures and chemical profiling of the extract. Various receptor targets can be selected to determine the therapeutic potential of *R. glutinosa*. Well-organized in vitro and in vivo assays will also help determine the potential of the plant to serve as a therapeutic agent for the treatment of metabolic diseases, such as T2DM and obesity.

## 4. Materials and Methods

### 4.1. General Experimental Procedures

Optical rotation data and ultraviolet (UV) spectra were obtained on a JASCO P-2000 polarimeter (Tokyo, Japan) and a Hitachi U-3000 UV/visible-light spectrophotometer (Tokyo, Japan), respectively. Infrared spectra were recorded on a Nicolet iS10 FT-IR spectrometer (Thermo Fisher, Waltham, MA, USA). NMR spectra were recorded on an Agilent 400 MHz Fourier-transform NMR instrument (Agilent Technologies, Santa Clara, CA, USA) and analyzed in MestreNova 9.0.0 software (Mestrelab Research S.L., Santiago de Compostela, Spain). HR-ESIMS was acquired on an Agilent 6230 time-of-flight liquid chromatography (LC)/mass spectrometer (Agilent Technologies). Adsorption column chromatography was performed using a silica gel (63–200 μm, Merck, Darmstadt, Germany). Medium-pressure LC was chromatographed on a CombiFlash Rf-200 instrument (Teledyne Isco, Lincoln, NE, USA), and RediSep Silver Silica Gel Disposable Flash Columns of 330.0 g and 24.0 g (Teledyne Isco) were used for the separations. Preparative high-performance liquid chromatography (MPLC) was carried out on a YMC-Pack Pro C 18 column (20 × 250 mm, 5 μm, YMC Co., Kyoto, Japan) using a Waters 600 pump and a Waters 996 photodiode array detector (Waters, Milford, MA, USA).

### 4.2. Plant Material

Dried roots of *R. glutinosa* were purchased from the Nonglim Saengyak Company in Seoul, South Korea, in January 2020. A voucher specimen (no. EA388) was deposited at the Natural Product Chemistry Laboratory, College of Pharmacy, Ewha Womans University.

### 4.3. Extraction and Isolation

The ground root of *R. glutinosa* (9 kg) was extracted with MeOH (13 × 5 L) by maceration and dried under reduced pressure to produce 987 g of crude extract. This extract was partitioned with hexanes (10 L, 16 g), EtOAc (10 L, 9 g), and n-BuOH (80 L, 90 g) sequentially.

The ethyl acetate soluble layer was fractionated on a silica gel column (CH_2_Cl_2_-MeOH) to produce 10 fractions (E01–E10). Fractions E04 and E05 (530 mg) were loaded together on an MPLC column to produce 10 subfractions (E0401–E0410). Among these, E0403 (30 mg) was purified using a silica gel to produce 15 subfractions (E040301–E040315). Of these, subfraction E040305 (10.5 mg) was chromatographed using semi-preparative reverse phase (RP) C-18 columns with MeOH-H_2_O (55:45) used as a solvent to yield compounds **2** (2.9 mg, t_R_ = 37.7 min), **6** (2.0 mg, t_R_ = 58.9 min), and **7** (0.7 mg, t_R_ = 63.4 min). Subfraction E040309 (125 mg) was purified in a semi-preparative RP C-18 column using MeCN-H_2_O (47:53) as a solvent to produce compound **3** (17.3 mg, t_R_ = 102.7 min). Subfraction E06 was subjected to an MPLC column (CH_2_Cl_2_-MeOH) to yield eight subfractions (E0601–E0608). Subfraction E0608 (991 mg) was separated on a silica gel column (hexane-Acetone) to produce 10 fractions (E060801–E060810), followed by purification of E060807 (55 mg) over a semi-preparative RP C-18 column (MeOH-H_2_O, 10 to 100) to produce compound **8** (0.9 mg, t_R_ = 19.6 min). Fraction E08 (1.8 g) was chromatographed on a MPLC column (CH_2_Cl_2_-MeOH) to yield 19 subfractions (E0801–E0819). Subfraction E0808 (249 mg) was eluted on an MPLC column (CH_2_Cl_2_-acetone, 100% to 0%) to afford nine subfractions (E0808701–E080809). Fraction E080806 was purified using a semi-preparative RP C-18 column, with MeOH-H_2_O (75:25) to produce compound **9** (11.3 mg, t_R_ = 40.0 min). E0811 was fractionated using a semi-preparative RP C-18 column (MeOH-H_2_O, 65:35) into four subfractions (E081101–E081104). Subfraction E081102 was purified on ODS-A and semi-preparative (MeOH-H_2_O, 45:55) C-18 columns to afford compounds **4** (0.9 mg, t_R_ = 58.2 min), **18** (16.7 mg, t_R_ = 25.8 min), and **20** (17.6 mg, t_R_ = 32.7 min). Subfraction E0814 (260 mg) was chromatographed on an RP C-18 column to produce 19 subfractions (E081401–E081419). Among these, E081401 was subjected to purification over a semi-preparative RP C-18 column using MeOH-H_2_O (25:75) as a solvent to produce compounds **1** (1.7 mg, t_R_ = 20.3 min) and **14** (8.2 mg, t_R_ = 45.4 min). E081402 and E081404 were then subjected to a semi-preparative RP C-18 column (MeOH-H_2_O, 25:75) to produce compounds **11** (1.7 mg, t_R_ = 45.7 min) and **5** (13.1 mg, t_R_ = 36.1 min), respectively. E081403 was chromatographed over a Sephadex-LH20, and compound **17** (30.5 mg) was precipitated from one of the subfractions. Fraction E09 (2.2 g) was chromatographed on a MPLC column to yield 12 subfractions E0901–E0912). One of these fractions, E0906 (330 mg), was then subjected to Sephadex LH-20 and a semi-preparative RP C-18 column (CH_3_CN-H_2_O, 60:40) chromatography to afford compound **13** (35 mg, t_R_ = 37.2 min).

The n-butanol soluble layer was loaded on a silica gel column (CH_2_Cl_2_-MeOH) to fractionate into 12 fractions (B01–B12), and compounds **10** (445 mg) and **21** (586 mg) were precipitated from fractions B08 and B09, respectively. B07 (39 g) was chromatographed on a silica gel (CH_2_Cl_2_-MeOH-H_2_O and 100% MeOH) to produce 12 subfractions (B0701–B0712). Subfraction B0710 (9.1 g) was subjected to an octadecylsilyl (ODS) column (MeOH-H_2_O, 0–100%) to yield 21 subfractions (B071001–B071021). Subsequent separation of B071015 (108 mg) over a semi-preparative RP C-18 column using MeOH-H_2_O (32:68) as the solvent yielded compounds **15** (20.5 mg, t_R_ = 97.4 min) and **16** (22.4 mg, t_R_ = 46.9 min). Compound **19** (10.2 mg, t_R_ = 68.6 min) was purified from B071018 on a semi-preparative RP C-18 column (MeOH-H_2_O, 35:65). Subfraction B071011 (106 mg) was followed up by charging onto an ODS column (MeOH-H_2_O, 10–100%) to afford 11 subfractions (B07101101–B07101111). Subfraction B07101106 (35.0 mg) was eluted on a silica gel to yield 10 subfractions (B0710110601–B0710110610), followed by further separation of B0710110608 (20.4 mg) over a semi-preparative RP C-18 column (MeOH-H_2_O, 40%), to yield compound **12** (6.2 mg, t_R_ = 164.2 min).

1-*O*-Ethyl-β-D-glucopyranosyl-(6 → 3′)-uridine (compound **1**): Brown gum; [α]^20^_D_ -8.0 (c 0.5,MeOH); UV (MeOH) λ_max_ (log ε) 262 (2.78), 201 (2.88) nm; IR (KBr) ν_max_ 3587, 2962, 1647, 1260, 1031 cm^−1^; ^1^H NMR (CD_3_OD, 400 MHz) δ_H_ 5.69 (1H, d, *J* = 8.0 Hz, H-5), 8.00 (1H, d, *J* = 8.0 Hz, H-6), 5.90 (1H, d, *J* = 4.7 Hz, H-1′), 4.17 (1H, ddd, *J*_1′,2′_ = 4.7 Hz, *J*_2′,3′_ = 4.6 Hz, H-2′), 4.15 (1H, ddd, *J*_2′,3′_ = 4.6 Hz, *J*_3′,4′_ = 4.5 Hz, H-3′), 4.00 (1H, dt, *J* = 4.5, 3.0 Hz, H-4′), 3.73 (1H, dd, *J* = 12.2, 3.0 Hz, H-5′a), 3.83 (1H, dd, *J* = 12.2, 3.0 Hz, H-5′b), 4.26 (1H, d, *J* = 7.8 Hz, H-1″), 3.16 (1H, dd, *J* = 9.2, 7.8 Hz, H-2″), 3.34 (1H, d, *J* = 2.0 Hz, H-3″), 3.27 (1H, d, *J* = 2.0 Hz, H-4″), 3.26 (1H, dt, *J* = 2.1, 0.8 Hz, H-5″), 3.66 (1H, dd, *J* = 12.0, 5.4 Hz, H-6″a), 3.86 (1H, dd, *J* = 12.0, 2.1 Hz, H-6′’b), 3.61 (1H, dd, *J* = 9.5, 6.9 Hz, 1″-O-CH_2_CH_3_), 3.96 (1H, dd, *J* = 9.5, 6.9 Hz, 1″-O-CH_2_CH_3_), 1.23 (3H, t, *J* = 6.9 Hz, 1″-O-CH_2_CH_3_);^13^C NMR (CD_3_OD, 100 MHz) δ_C_ 152.5 (C-2), 166.3 (C-4), 102.7 (C-5), 142.8 (C-6), 90.8 (C-1′), 75.8 (C-2′), 71.4 (C-3′), 86.4 (C-4′), 62.3 (C-5′), 104.2 (C-1″), 75.2 (C-2″), 78.2 (C-3″), 71.7 (C-4″), 78.0 (C-5″), 62.8 (C-6″), 66.2 (1″-O-CH_2_CH_3_), 15.5 (1″-O-CH_2_CH_3_); HR-ESIMS m/z 236.1232 [M + 2H_3_O]^2+^ (calculated for C_17_H_32_N_2_O_13_, 472.1893); CD (MeOH) *λ* (Δ*ε*) 220 (−2.99), 243 (−3.09), 273 (+5.47) nm.

1,8-Dihydroxy-2,2,8-trimethyl-6-oxabicyclo[3.2.1]octane-7-carboxylic acid (compound **2**): amorphous white powder; [α]^20^_D_ +52.0 (c 0.5,MeOH); UV (MeOH) λ_max_ (log ε) 196 (3.12) nm; IR (KBr) 3401, 2966, 1772, 997 cm^−1^; ^1^H NMR (CD_3_OD, 400 MHz) δ_H_ 1.37(1H, dddd, *J* = 11.4, 6.7, 2.1 Hz, H-3a), 1.93(1H, m, H-3b), 1.82(1H, m, H-4a), 1.90(1H, m, H-4b), 4.06(1H, d, *J* = 5.6 Hz, H-5), 4.20(1H, s, H-7), 1.52(3H, s, H-10), 1.04(3H, s, H-11), 1.05(3H, s, H-12); ^13^C NMR (CD_3_OD, 100 MHz) δ_C_ 85.8 (C-1), 38.6 (C-2), 33.6 (C-3), 25.6 (C-4), 81.9 (C-5), 81.4 (C-7), 92.1 (C-8), 174.8 (C-9), 13.3 (C-10), 24.4 (C-11), 27.9 (C-12); HR-ESIMS m/z 253.1051 [M + Na]^+^ (calculated for C_11_H_18_O_5_Na, 253.1046); CD (MeOH) *λ* (Δ*ε*) 199 (+15.18), 228 (+33.33) nm.

2′-Hydroxyethyl-(2*E*,4*E*)-2-methyl-6-oxohepta-2,4-dienoate (compound **8**): amorphous white powder; UV (MeOH) λ_max_ (log ε) 279 (3.07) nm; IR (KBr) ν_max_ 3361, 1647, 1260 cm^−1^; ^1^H NMR (CD_3_OD, 400 MHz) δ_H_ 6.92 (1H, ddd, *J* = 11.5,1.4,0.8 Hz, H-3), 7.56 (1H, dd, *J* = 15.6, 11.5 Hz, H-4), 6.38 (1H, d, *J* = 15.6 Hz, H-5), 2.33 (3H,s, H-7), 2.11 (3H, d, *J* = 0.8 Hz, H-8), 3.39 (2H, t, *J* = 6.0 Hz, H-1′), 3.64 (2H, t, *J* = 6.0 Hz, H-2″); ^13^C NMR (CD_3_OD, 100 MHz) δ_C_ 171.4 (C-1), 141.4 (C-2), 131.7 (C-3), 139.1 (C-4), 135.1 (C-5), 201.1 (C-6), 27.6 (C-7), 13.8 (C-8), 43.5 (C-1′), 61.5 (C-2′); HR-ESIMS m/z 100.0519 [M + 2H]^2+^ (calculated for C_10_H_16_O_4_, 200.1038).

### 4.4. In Silico Simulation

#### 4.4.1. Molecular Docking

The molecular docking analysis began by obtaining the 3D structure of AMPK from the RCSB Protein Data Bank database (https://www.rcsb.org/, accessed on 15 November 2024) using the identifier 5ISO. The 3D structures of **1**–**21** were drawn using ChemSketch (ACD/Labs, Toronto, ON, Canada) and optimized with its integrated 3D optimization tool. AICAR, serving as a positive control, was obtained from the PubChem database (https://pubchem.ncbi.nlm.nih.gov, accessed on 15 November 2024). All ligands were subsequently converted to appropriate file formats (pdb and mol2) using Open Babel version 3.1.1 (https://openbabel.org/index.html, accessed on 15 November 2024). Using UCSF Chimera (UCSF, San Francisco, CA, USA), both AMPK and the ligands underwent preparation processes, including hydrogen addition and charge assignment. The docking simulation employed four different programs: Autodock Vina 1.1.2 (Scripps Research, San Diego, CA, USA), Autodock 4.2.6 (Scripps Research, CA, USA), and Dock6 (UCSF, CA, USA). The size of the grid box used was 15 × 15× 15 for Autodock Vina and 40 × 40 × 40 for Autodock4. The grid size of Dock6 was not defined due to an automatic clustering function of Dock6 during the generation of the grid. The binding-site and grid-box dimensions were determined based on the co-crystallized ligand’s coordinates (x: −3.465, y: −56.795, z: 35.109), following each software’s specific guidelines. Following the docking simulations, a pharmacophore analysis was conducted using LigandScout 4.0 (inte: Ligand, Vienna, Austria) to investigate potential interactions between the receptors and ligands.

#### 4.4.2. Molecular Dynamics

Molecular dynamics (MD) simulations were performed using GROMACS 2024.3 (https://www.gromacs.org, accessed on 24 October 2024). The preparation of the AMPK–compound **1** complex involved separate preprocessing of the protein and ligand structures, with ligand topology generated through the ATB server (https://atb.uq.edu.au, accessed on 11 December 2024). The ligand topology was then merged with the protein topology processed using the 54a7 force field. The complex was situated in a cubic box with a 1.0 nm buffer and solvated using simple point-charge water. After energy minimization using the steepest descent algorithm, the system underwent equilibration in two stages: 100 ps NVT at 300 K followed by 100 ps NPT at 1 bar. The production MD simulations ran for 100 ns using a 2 fs time step, incorporating periodic boundary conditions and the particle-mesh Ewald method for long-range electrostatics calculations. The resulting trajectories were analyzed using GROMACS tools to calculate RMSD, RMSF, and hydrogen bond interactions.

#### 4.4.3. ADMET Prediction

The prediction of ADMET properties began by preparing the ligand structure of compound **1** in the SMILES format using ChemSketch, which was then uploaded to ADMETlab2.0 (https://admetmesh.scbdd.com, accessed on 22 October 2024) and SwissADME (http://www.swissadme.ch, accessed on 22 October 2024) web servers. The analysis encompassed various absorption properties, including water solubility, Caco-2 permeability, and gastrointestinal absorption. The distribution assessment focused on the volume of distribution and blood–brain barrier penetration, while metabolism predictions examined the cytochrome P450 enzyme inhibition profiles for five key enzymes: CYP1A2, CYP2C9, CYP2C19, CYP2D6, and CYP3A4. The excretion analysis used predicted clearance rates, and the toxicity evaluations included assessments of hepatotoxicity, hERG inhibition, and mutagenicity through the AMES test. All predictions were conducted using default parameters provided by both platforms.

### 4.5. In Vitro Assay

#### 4.5.1. Cell Treatment Experiments

The experiments were performed based on our previous study [47]. HepG2 cells, a human hepatocellular carcinoma line, were sourced from the American Type Culture Collection (Manassas, VA, USA). The cells were grown in Dulbecco’s modified Eagle medium (Welgene, Gyeongsan, Republic of Korea), supplemented with 10% fetal bovine serum inactivated at 56 °C for 30 min (Welgene), along with 100 U/mL penicillin and 100 μg/mL streptomycin (Welgene). The cultures were maintained in a humidified incubator at 37 °C with 5% CO_2_. The medium was replaced every two days to eliminate non-adherent cells and debris. To evaluate the effects of compound **1** on the activation of AMPK, HepG2 cells were treated with 5, 10, and 20 μM of **1** and 200 and 400 μM of AICAR for 2 h. In the next round, the cells were treated with 2, 4, and 8 μM of **1** and 500 μM of AICAR for 12 h.

#### 4.5.2. Cell Lysis and Protein Extraction

Cells were washed with phosphate-buffered saline (PBS, Gibco, Grand Island, NY, USA) and collected using chilled PBS to lyse them and extract proteins. Protein extraction was performed with a cold RIPA buffer (Biosesang, Seongnam, Republic of Korea) supplemented with a protease inhibitor cocktail (GenDEPOT, Katy, TX, USA), following the manufacturer’s guidelines. Whole-cell lysates were prepared by heating 10–40 μg of total protein at 98 °C for 5 min in a gel-loading buffer (0.3125 M Tris-HCl pH 6.8, 2% SDS, 5% 2-mercaptoethanol, 0.05% bromophenol blue, and 25% glycerol) at a 4:1 ratio. Protein quantification was achieved with Pierce BCA Protein Assay Kits (Thermo Scientific, Waltham, MA, USA). Sodium dodecyl sulfate–polyacrylamide gel electrophoresis was used to separate proteins, which were then transferred onto polyvinyl difluoride membranes (Millipore) using a Bio-Rad Western system. The membranes were blocked with 5% non-fat milk in TBS (50 mM Tris, 150 mM NaCl, pH 7.6) containing 0.1% Tween 20 (TBS-T) for 1 h and then washed five times for 10 min each in TBS-T. The membranes were incubated overnight at 4 °C with primary antibodies diluted to 1:1000. The following day, they were washed five times for 15 min each in TBS-T and then treated for 1 h at 25 °C with secondary antibodies conjugated to horseradish peroxidase (HRP) (1:10,000). Finally, immunoblots were visualized using a chemiluminescent HRP substrate (Advansta, San Jose, CA, USA) and a ChemiDoc imaging system (Bio-Rad, Hercules, CA, USA), according to the manufacturer’s instructions.

#### 4.5.3. Reagents

All antibodies were purchased from Santa Cruz Biotechnology (Dallas, TX, USA) and Cell Signaling Technology, Inc. (Beverly, MA, USA). The primary antibodies used were anti-GAPDH (sc-25778) and anti-AMPK (Thr172) (#2535). AICAR (A9978), an AMPK activator, was obtained from Sigma-Aldrich (Milwaukee, WI, USA).

## 5. Conclusions

In this study, 21 compounds were isolated from the dried roots of *R. glutinosa*, and 3 of them (compounds **1**, **2**, and **8**) were newly found structures in nature. These were uridine glycoside, *O*-bridged bicyclic skeleton, and hepta-dienoate skeletons, respectively. Compound **9** was found for the first time in this plant. In silico docking simulations suggested that compound **1** can be a strong AMPK activator, and this finding was supported by an in vitro assay. This study provided new structures that can be found in *R. glutinosa*, and we also found a potential ingredient (compound **1**) that is active in AMPK activation. Future studies should investigate the mechanisms of action of compound **1** in the AMPK-signaling pathway and concentration–activity relationship of compound **1** with AMPK. Therefore, RNA-related assay and metabolomics study will be performed, followed by T2DM/obesity mouse model experiments.

## Figures and Tables

**Table 1:** Docking energy (Kcal/mol) of compounds **1**–**21** and AICAR with AMPK.

Compound	Autodock Vina	Autodock4	Dock6
AICAR *	−6.4	−6.9	−37.3
**1**	−6	−8.2	−58.4
**2**	−4.7	−4.8	−25.5
**3**	−4.8	−5.2	−27.6
**4**	−4.6	−6.8	−43.7
**5**	−5.5	−7.9	−44.6
**6**	−5.1	−6.2	−29.0
**7**	−4.4	−6.6	−26.2
**8**	−5.3	−5.5	−29.8
**9**	−6.5	−8.4	−44.0
**10**	−6.8	−9.3	−48.3
**11**	−6.3	−9.7	−50.1
**12**	−5.9	−1.4	−57.8
**13**	−4.1	−10.5	−53.7
**14**	−6.9	−7.9	−39.4
**15**	−5.5	−6.6	−66.8
**16**	−4.6	3.6	−71.0
**17**	−5.3	−8.7	−64.4
**18**	−4.7	−2.1	−65.2
**19**	−2.6	13.2	−68.8
**20**	−6.2	−10.0	−67.9
**21**	−6.1	−11.5	−38.4

* AICAR, 5-amino-4-imidazolecarboxamide ribonucleoside.

## Data Availability

The data will be made available on request.

## Supplementary Materials

The following supporting information can be downloaded at https://www.mdpi.com/article/10.3390/molecules29246009/s1. Figure S1. UV spectrum of **1**; Figure S2. HRESIMS spectrum of **1**; Figure S3. ^1^H NMR spectrum of **1** (400 MHz, CD_3_OD); Figure S4. ^13^C NMR spectrum of **1** (100 MHz, CD_3_OD); Figure S5. DEPT-135 NMR spectrum of **1** (100 MHz, CD_3_OD); Figure S6. ^1^H-^13^C HSQC NMR spectrum of **1** (CD_3_OD); Figure S7. ^1^H-^13^C HMBC NMR spectrum of **1** (CD_3_OD); Figure S8. ^1^H-^1^H COSY NMR spectrum of **1** (CD_3_OD); Figure S9. ^1^H-^1^H NOESY NMR spectrum of **1** (CD_3_OD); Figure S10. CD spectrum of **1**; Figure S11. UV spectrum of **2**; Figure S12. HRESIMS spectrum of **2**; Figure S13. ^1^H NMR spectrum of **2** (400 MHz, CD_3_OD); Figure S14. ^13^C NMR spectrum of **2** (100 MHz, CD_3_OD); Figure S15. DEPT-135 NMR spectrum of **2** (100 MHz, CD_3_OD); Figure S16. ^1^H-^13^C HSQC NMR spectrum of **2** (CD_3_OD); Figure S17. ^1^H-^13^C HMBC NMR spectrum of **2** (CD_3_OD); Figure S18. ^1^H-^1^H COSY NMR spectrum of **2** (CD_3_OD); Figure S19. ^1^H-^1^H NOESY NMR spectrum of **2** (CD_3_OD); Figure S20. UV spectrum of **8**; Figure S21. HRESIMS spectrum of **8**; Figure S22. ^1^H NMR spectrum of **8** (400 MHz, CD_3_OD); Figure S23. ^13^C NMR spectrum of **8** (100 MHz, CD_3_OD); Figure S24. DEPT-135 NMR spectrum of **8** (100 MHz, CD_3_OD); Figure S25. ^1^H-^13^C HSQC NMR spectrum of **8** (CD_3_OD); Figure S26. ^1^H-^13^C HMBC NMR spectrum of **8** (CD_3_OD); Figure S27. ^1^H-^1^H COSY NMR spectrum of **8** (CD_3_OD); Figure S28. ^1^H-^1^H NOESY NMR spectrum of **8** (CD_3_OD); Table S1. In silico physicochemical and ADMET profiling of compound **1**.

## References

[1] Li X.-J., Jiang C., Xu N., Li J.-X., Meng F.-Y., Zhai H.-Q. (2018). Sorting and identification of *Rehmannia glutinosa* germplasm resources based on EST-SSR, scanning electron microscopy micromorphology, and quantitative taxonomy. Ind. Crops Prod..

[2] Cao Q., Wang Z., Jiang Y., Dong C. (2024). *Rehmannia glutinosa* polysaccharides: A review on structure-activity relationship and biological activity. Med. Chem. Res..

[3] Zhang R.-X., Li M.-X., Jia Z.-P. (2008). *Rehmannia glutinosa*: Review of botany, chemistry and pharmacology. J. Ethnopharmacol..

[4] Poon T.Y.C., Ong K.L., Cheung B.M.Y. (2011). Review of the effects of the traditional Chinese medicine *Rehmannia* Six Formula on diabetes mellitus and its complications. J. Diabetes.

[5] Han K., Bose S., Kim Y.-M., Chin Y.-W., Kim B.-S., Wang J.-H., Lee J.-H., Kim H. (2015). *Rehmannia glutinosa* reduced waist circumferences of Korean obese women possibly through modulation of gut microbiota. Food Funct..

[6] Kim S.-H., Yook T.-H., Kim J.-U. (2017). Rehmanniae Radix, an effective treatment for patients with various inflammatory and metabolic diseases: Results from a review of Korean publications. J. Pharmacopunct..

[7] Jiang L., Zhang N.-X., Mo W., Wan R., Ma C.-G., Li X., Gu Y.-L., Yang X.-Y., Tang Q.-Q., Song H.-Y. (2008). *Rehmannia* inhibits adipocyte differentiation and adipogenesis. Biochem. Biophys. Res. Commun..

[8] Park M.-Y., Lee H.J., Choi D.H., Kang B.-J., Choi S., Park Y.S. (2017). Oral administration of *Rehmannia glutinosa* extract for obesity treatment via adiposity and fatty acid binding protein expression in obese rats. Toxicol. Environ. Health Sci..

[9] Clemente-Suárez V.J., Martín-Rodríguez A., Redondo-Flórez L., López-Mora C., Yáñez-Sepúlveda R., Tornero-Aguilera J.F. (2023). New insights and potential therapeutic interventions in metabolic diseases. Int. J. Mol. Sci..

[10] Kazibwe J., Tran P.B., Annerstedt K.S. (2021). The household financial burden of non-communicable diseases in low-and middle-income countries: A systematic review. Health Res. Policy Syst..

[11] Atanasov A.G., Zotchev S.B., Dirsch V.M., Supuran C.T. (2021). Natural products in drug discovery: Advances and opportunities. Nat. Rev. Drug Discov..

[12] Hegde P.K., Rao H.A., Rao P.N. (2014). A review on Insulin plant (*Costus igneus* Nak). Phcog. Rev..

[13] Youn I., Piao D., Park J., Ock S.A., Han S., Han A.-R., Shin S., Seo E.K. (2024). Anti-obesity ativities of the compounds from *Perilla frutescens* var. *acuta* and chemical profiling of the extract. Molecules.

[14] Kim J., Yang G., Kim Y., Kim J., Ha J. (2016). AMPK activators: Mechanisms of action and physiological activities. Exp. Mol. Med..

[15] Mohseni R., Teimouri M., Safaei M., Arab Sadeghabadi Z. (2023). AMP-activated protein kinase is a key regulator of obesity-associated factors. Cell Biochem. Funct..

[16] Kakoti B.B., Alom S., Deka K., Halder R.K. (2024). AMPK pathway: An emerging target to control diabetes mellitus and its related complications. J. Diabetes Metab. Disord..

[17] Tripathi A., Misra K. (2017). Molecular docking: A structure-based drug designing approach. JSM Chem..

[18] Tuan C.D., Ngan T.B., Huong D.T.M., Quyen V.T., Murphy B., Van Minh C., Van Cuong P. (2017). Secondary metabolites from *Micromonospora* sp.(G044). J. Sci. Technol..

[19] Walczak D., Sikorski A., Grzywacz D., Nowacki A., Liberek B. (2022). Characteristic ^1^H NMR spectra of β-D-ribofuranosides and ribonucleosides: Factors driving furanose ring conformations. RSC Adv..

[20] Saimaru H., Orihara Y. (2010). Biosynthesis of acteoside in cultured cells of *Olea europaea*. J. Nat. Med..

[21] Miyahara T., Nakatsuji H., Wada T. (2014). Circular dichroism spectra of uridine derivatives: ChiraSac study. J. Phys. Chem. A.

[22] Wenkert E., Guo M., Lavilla R., Porter B., Ramachandran K., Sheu J.H. (1990). Polyene synthesis. Ready construction of retinol-carotene fragments,(±)-6(*E*)-LTB_3_ leukotrienes, and corticrocin. J. Org. Chem..

[23] Endo T., Taguchi H., Sasaki H., Yosioka I. (1979). Studies on the constituents of *Aeginetia indica* L. var. *gracilis* Nakai. Structures of three glycosides isolated from the whole plant. Chem. Pharm. Bull..

[24] Yoshikawa M., Fukuda Y., Taniyama T., Kitagawa I. (1996). Chemical studies on crude drug processing IX. On the constituents of Rehmanniae Radix (3) Absolute stereostructures of rehmaionosides A, B, and C, and rehmapicroside, biologically active ionone glucosides and a monoterpene glucoside isolated from Chinese Rehmanniae Radix. Chem. Pharm. Bull..

[25] Chen X., Cao Y.-G., Ren Y.-J., Liu Y.-L., Fan X.-L., He C., Ma X.-Y., Zheng X.-K., Feng W.-S. (2022). Ionones and lignans from the fresh roots of *Rehmannia glutinosa*. Phytochemistry.

[26] Sang S., Lao A., Wang Y., Chin C.-K., Rosen R.T., Ho C.-T. (2002). Antifungal constituents from the seeds of *Allium fistulosum* L.. J. Agric. Food Chem..

[27] Thao T.T.P., Bui T.Q., Quy P.T., Bao N.C., Van Loc T., Van Chien T., Chi N.L., Van Tuan N., Van Sung T., Nhung N.T.A. (2021). Isolation, semi-synthesis, docking-based prediction, and bioassay-based activity of *Dolichandrone spathacea* iridoids: New catalpol derivatives as glucosidase inhibitors. RSC Adv..

[28] Ono M., Ueno M., Masuoka C., Ikeda T., Nohara T. (2005). Iridoid glucosides from the fruit of *Genipa americana*. Chem. Pharm. Bull..

[29] Feng W.-S., Li M., Zheng X.-K., Zhang N., Song K., Wang J.-C., Kuang H.-X. (2015). Two new ionone glycosides from the roots of *Rehmannia glutinosa* Libosch. Nat. Prod. Res..

[30] Sasaki H., Nishimura H., Morota T., Katsuhara T., Chin M., Mitsuhashi H. (1991). Norcarotenoid glycosides of *Rehmannia glutinosa* var. *purpurea*. Phytochemistry.

[31] Lee S.-Y., Yean M.-H., Kim J.-S., Lee J.-H., Kang S.-S. (2011). Phytochemical studies on Rehmanniae Radix. Korean J. Pharmacogn..

[32] Sasaki H., Nishimura H., Chin M., Mitsuhashi H. (1989). Hydroxycinnamic acid esters of phenethylalcohol glycosides from *Rehmannia glutinosa* var. *purpurea*. Phytochemistry.

[33] Miyase T., Koizumi A., Ueno A., Noro T., Kuroyanagi M., Fukushima S., Akiyama Y., Takemoto T. (1982). Studies on the acyl glycosides from *Leucoseptrum japonicum* (Miq.) Kitamura et Murata. Chem. Pharm. Bull..

[34] Sasaki H., Taguchi H., Endo T., Yosioka I., Higashiyama K., Otomasu H. (1978). The glycosides of *Martynia louisiana* Mill. A new phenylpropanoid glycoside, martynoside. Chem. Pharm. Bull..

[35] Calis I., Lahloub M.F., Rogenmoser E., Sticher O. (1984). Isomartynoside, a phenylpropanoid glycoside from *Galeopsis pubescens*. Phytochemistry.

[36] Yusuf N., Yusup S., Yiin C., Ratri P., Halim A., Razak N. (2021). Prediction of solvation properties of low transition temperature mixtures (LTTMs) using COSMO-RS and NMR approach. IOP Conf. Ser. Mater. Sci. Eng..

[37] Sargsyan K., Grauffel C., Lim C. (2017). How molecular size impacts RMSD applications in molecular dynamics simulations. J. Chem. Theory Comput..

[38] Ghahremanian S., Rashidi M.M., Raeisi K., Toghraie D. (2022). Molecular dynamics simulation approach for discovering potential inhibitors against SARS-CoV-2: A structural review. J. Mol. Liq..

[39] Bhattamisra S.K., Yap K.H., Rao V., Choudhury H. (2019). Multiple biological effects of an iridoid glucoside, catalpol, and its underlying molecular mechanisms. Biomolecules.

[40] Zhang W., Zhang F., Hu Q., Xiao X., Ou L., Chen Y., Luo S., Cheng Y., Jiang Y., Ma X. (2021). The emerging possibility of the use of geniposide in the treatment of cerebral diseases: A review. Chin. Med..

[41] Han J., Luo L., Wang Y., Wu S., Kasim V. (2022). Therapeutic potential and molecular mechanisms of salidroside in ischemic diseases. Front. Pharmacol..

[42] Xiao Y., Ren Q., Wu L. (2022). The pharmacokinetic property and pharmacological activity of acteoside: A review. Biomed. Pharmacother..

[43] Liu S., Cheng X., Li X.F., Kong Y., Jiang S., Dong C., Wang G. (2020). Design, microwave synthesis, and molecular docking studies of catalpol crotonates as potential neuroprotective agent of diabetic encephalopathy. Sci. Rep..

[44] Yang H., Zhai B., Wang M., Fan Y., Wang J., Cheng J., Zou J., Zhang X., Shi Y., Guo D. (2022). The influence of rhein on the absorption of rehmaionoside D: *In vivo*, *in situ*, *in vitro*, and *in silico* studies. J. Ethnopharmacol..

[45] Yan J., Wang C., Jin Y., Meng Q., Liu Q., Liu Z., Liu K., Sun H. (2018). Catalpol ameliorates hepatic insulin resistance in type 2 diabetes through acting on AMPK/NOX4/PI3K/AKT pathway. Pharmacol. Res..

[46] Li Y., Chen Q., Sun H.-J., Zhang J.-H., Liu X. (2024). The active ingredient catalpol in *Rehmannia glutinosa* reduces blood glucose in diabetic rats via the AMPK pathway. Diabetes Metab. Syndr. Obes..

[47] Kim S.M., Lee B., An H.J., Kim D.H., Park K.C., Noh S.-G., Chung K.W., Lee E.K., Kim K.M., Kim S.J. (2017). Novel PPARα agonist MHY553 alleviates hepatic steatosis by increasing fatty acid oxidation and decreasing inflammation during aging. Oncotarget.

