# Supplementary material for: Identification of Bioactive Compounds from the Roots of Rehmannia glutinosa and Their In Silico and In Vitro AMPK Activation Potential
Source: Molecules. 2024 Dec 20;29(24):6009. doi: 10.3390/molecules29246009 (PMC11679303; doi:10.3390/molecules29246009)
Supplement: Supplementary file 1 [file molecules-29-06009-s001.zip › molecules-3358471-supplementary.pdf]

Supplementary Materials

Identification of Bioactive Compounds from the Roots of *Rehmannia glutinosa* and Their *In Silico* and *In Vitro* AMPK Activation Potential

Hwaryeong Lee [1,†], Isoo Youn [1,†], Sang Gyun Noh [2], Hyun Woo Kim [2], Eunhye Song [1], Sang-Jip Nam [3], Hae Young Chung [2], and Eun Kyoung Seo [1,*]

[1] Graduate School of Pharmaceutical Sciences, College of Pharmacy, Ewha Womans University, Seoul 03760, Republic of Korea; jongsky119@naver.com (H.L.); iyoun@ewha.ac.kr (I.Y.); seh0413@ewhain.net (E.S.)
[2] Department of Pharmacy, College of Pharmacy, Pusan National University, Busan 46241, Republic of Korea; rskrsk92@pusan.ac.kr (S.G.N.); khw124124@naver.com (H.W.K.); hyjung@pusan.ac.kr (H.Y.C.)
[3] Department of Chemistry and Nanoscience, Ewha Womans University, Seoul 03760, Republic of Korea; sjnam@ewha.ac.kr
* Correspondence: yuny@ewha.ac.kr
† These authors equally contributed to this work.

Contents

Figure S1. UV spectrum of **1** -- 4

Figure S2. HRESIMS spectrum of **1** --- 4

Figure S3. ^{1}H NMR spectrum of **1** (400 MHz, CD_3OD) ------------------------------------ 5

Figure S4. ^{13}C NMR spectrum of **1** (100 MHz, CD_3OD) ------------------------------------ 5

Figure S5. DEPT-135 NMR spectrum of **1** (100 MHz, CD_3OD) --------------------------- 6

Figure S6. ^{1}H-^{13}C HSQC NMR spectrum of **1** (CD_3OD) ------------------------------------ 6

Figure S7. ^{1}H-^{13}C HMBC NMR spectrum of **1** (CD_3OD) ----------------------------------- 7

Figure S8. ^{1}H-^{1}H COSY NMR spectrum of **1** (CD_3OD) ------------------------------------- 7

Figure S9. ^{1}H-^{1}H NOESY NMR spectrum of **1** (CD_3OD) ----------------------------------- 8

Figure S10. CD spectrum of **1** -- 8

Figure S11. UV spectrum of **2** -- 9

Figure S12. HRESIMS spectrum of **2** -- 9

Figure S13. ^{1}H NMR spectrum of **2** (400 MHz, CD_3OD) ----------------------------------- 10

Figure S14. ^{13}C NMR spectrum of **2** (100 MHz, CD_3OD) ---------------------------------- 10

Figure S15. DEPT-135 NMR spectrum of **2** (100 MHz, CD_3OD) ------------------------- 11

Figure S16. ^{1}H-^{13}C HSQC NMR spectrum of **2** (CD_3OD) ---------------------------------- 11

Figure S17. ^{1}H-^{13}C HMBC NMR spectrum of **2** (CD_3OD) --------------------------------- 12

Figure S18. ^{1}H-^{1}H COSY NMR spectrum of **2** (CD_3OD) ----------------------------------- 12

Figure S19. ^{1}H-^{1}H NOESY NMR spectrum of **2** (CD_3OD) --------------------------------- 13

Figure S20. UV spectrum of **8** -- 13

Figure S21. HRESIMS spectrum of **8** -- 14

Figure S22. ^{1}H NMR spectrum of **8** (400 MHz, CD_3OD) ----------------------------------- 14

Figure S23. ^{13}C NMR spectrum of **8** (100 MHz, CD_3OD) ---------------------------------- 15

Figure S24. DEPT-135 NMR spectrum of **8** (100 MHz, CD_3OD) ------------------------- 15

Figure S25. ^{1}H-^{13}C HSQC NMR spectrum of **8** (CD_3OD) ---------------------------------- 16

Figure S26. ^{1}H-^{13}C HMBC NMR spectrum of **8** (CD_3OD) --------------------------------- 16

Figure S27. ^1H-^1H COSY NMR spectrum of **8** (CD_3OD) ------------------------------------ 17

Figure S28. ^1H-^1H NOESY NMR spectrum of **8** (CD_3OD) ---------------------------------- 17

Table S1. *In silico* physicochemical and ADMET profiling of compound **1** ---------------- 18

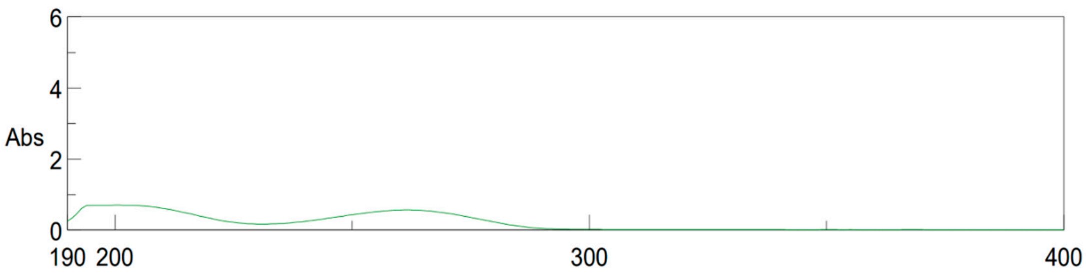

Figure S1. UV spectrum of **1**

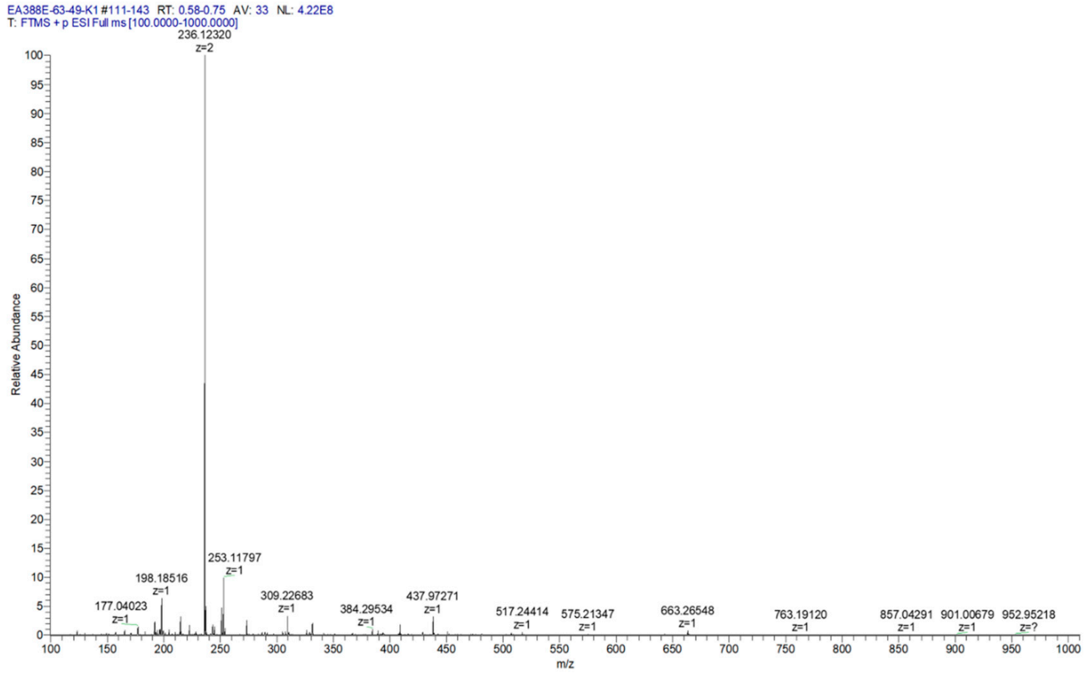

Figure S2. HRESIMS spectrum of **1**

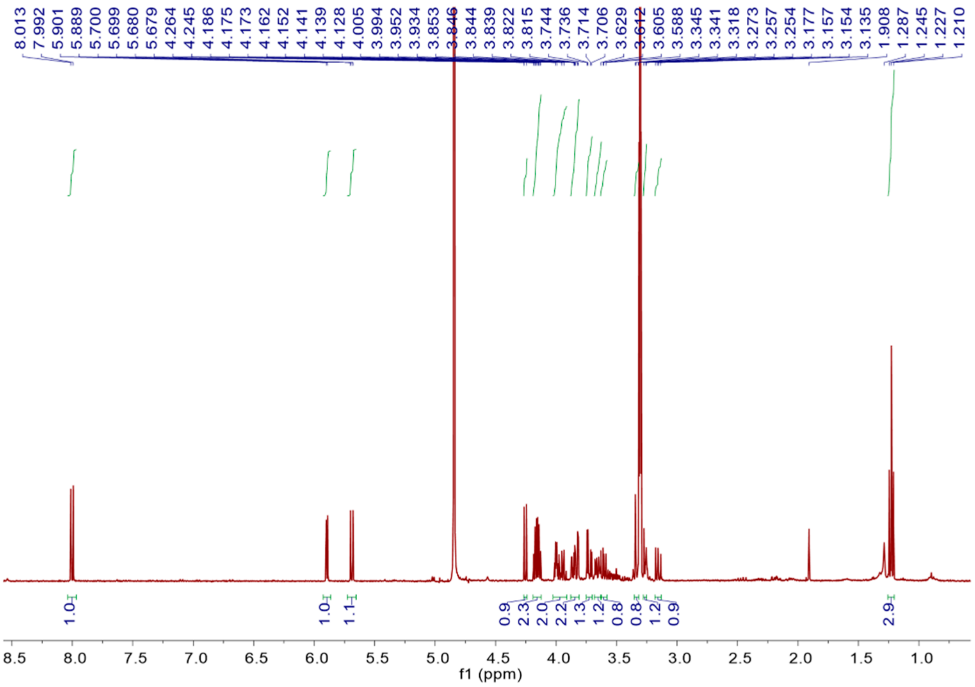

Figure S3. ^{1}H NMR spectrum of **1** (400 MHz, CD_3OD)

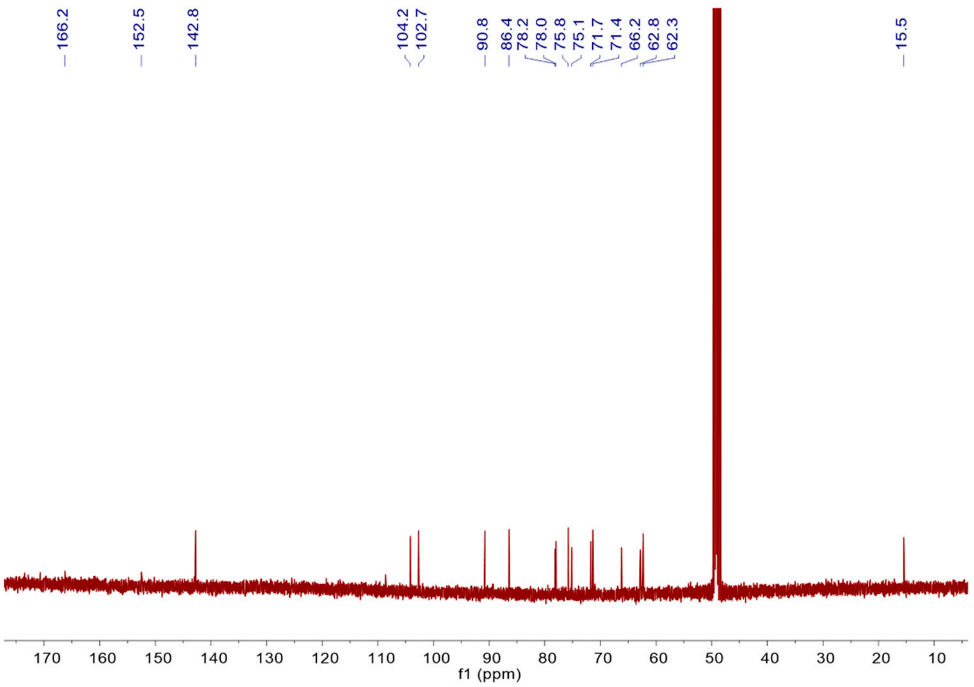

Figure S4. ^{13}C NMR spectrum of **1** (100 MHz, CD_3OD)

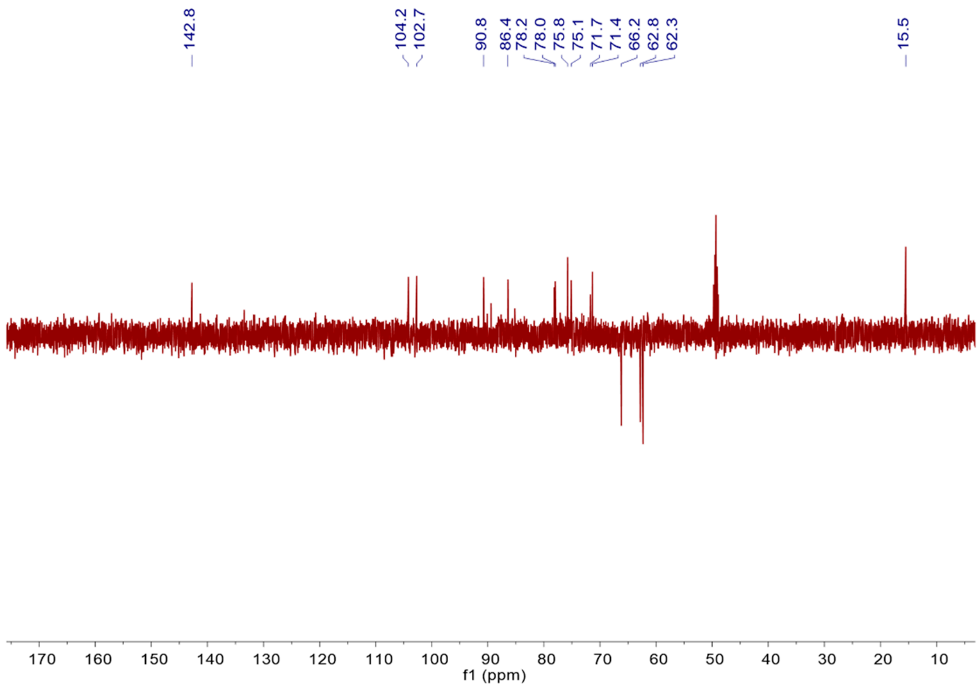

Figure S5. DEPT-135 NMR spectrum of **1** (100 MHz, CD_3OD)

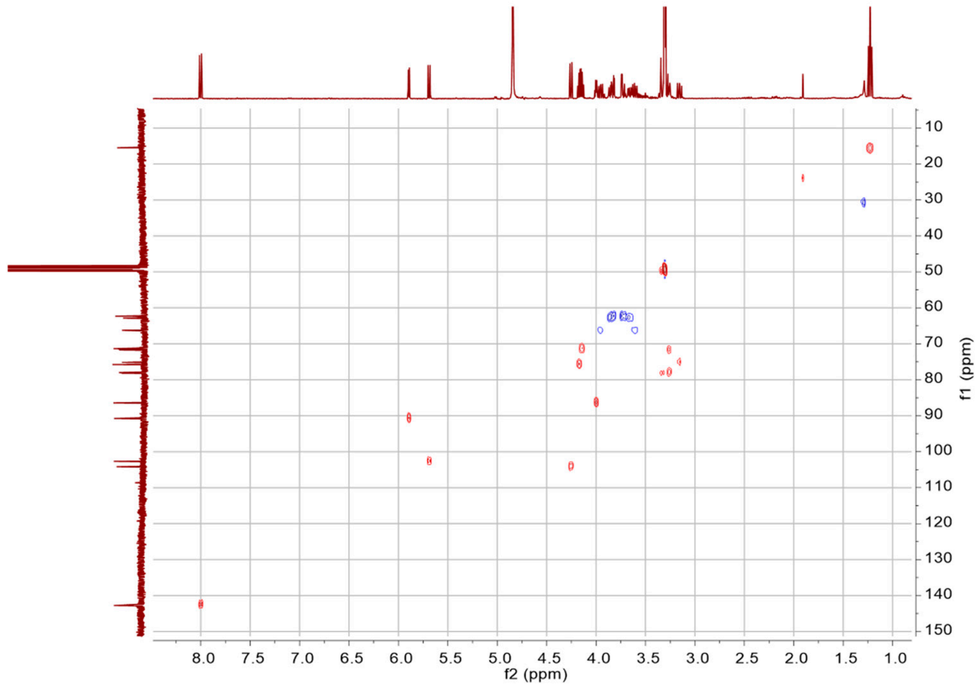

Figure S6. ^{1}H-^{13}C HSQC NMR spectrum of **1** (CD_3OD)

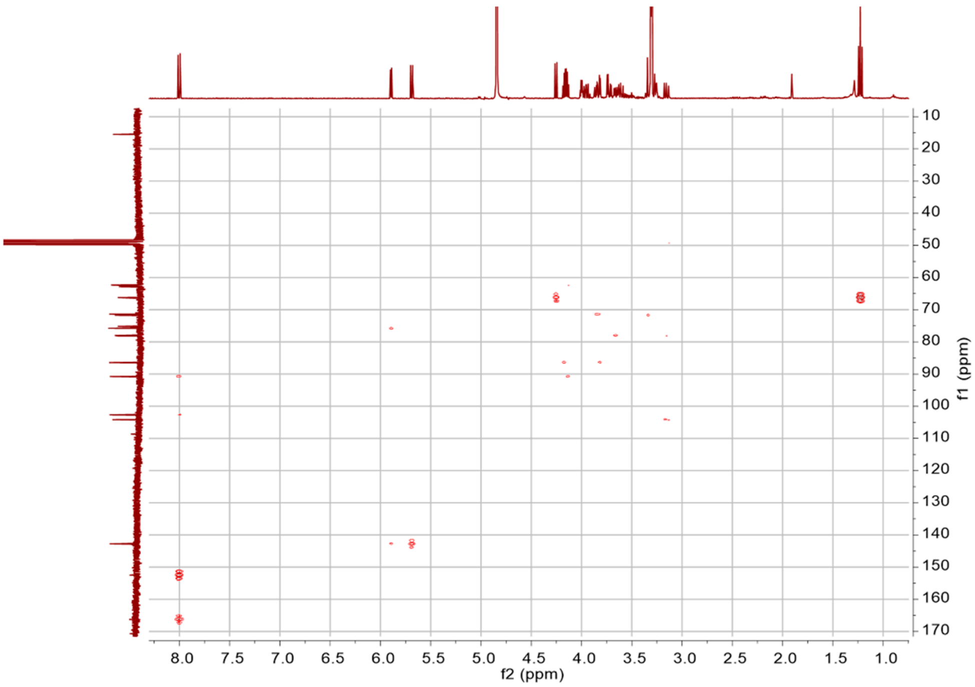

Figure S7. ^{1}H-^{13}C HMBC NMR spectrum of **1** (CD_3OD)

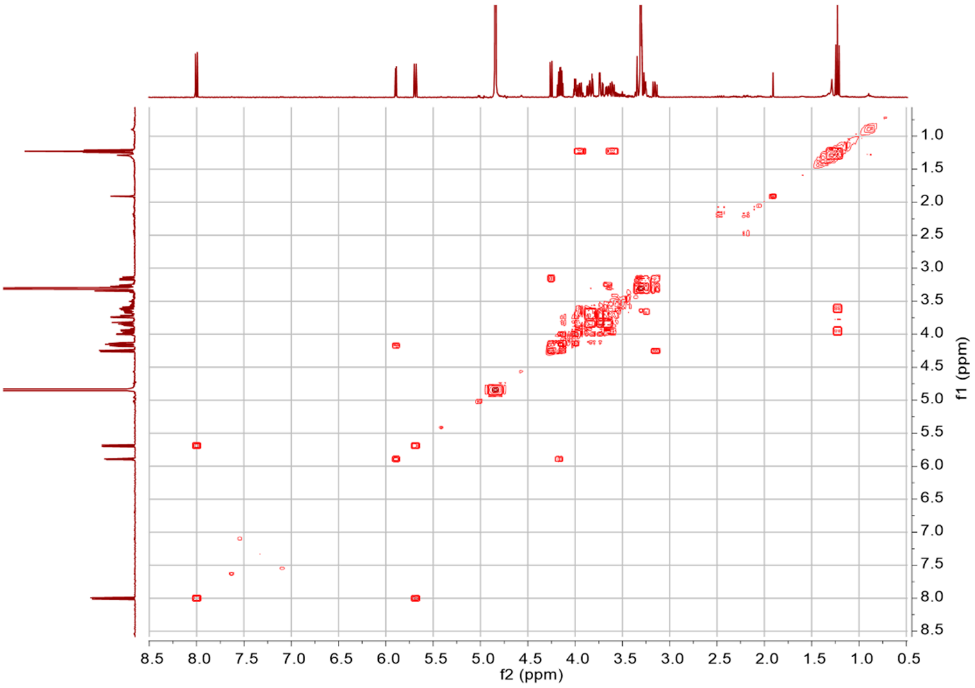

Figure S8. ^{1}H-^{1}H COSY NMR spectrum of **1** (CD_3OD)

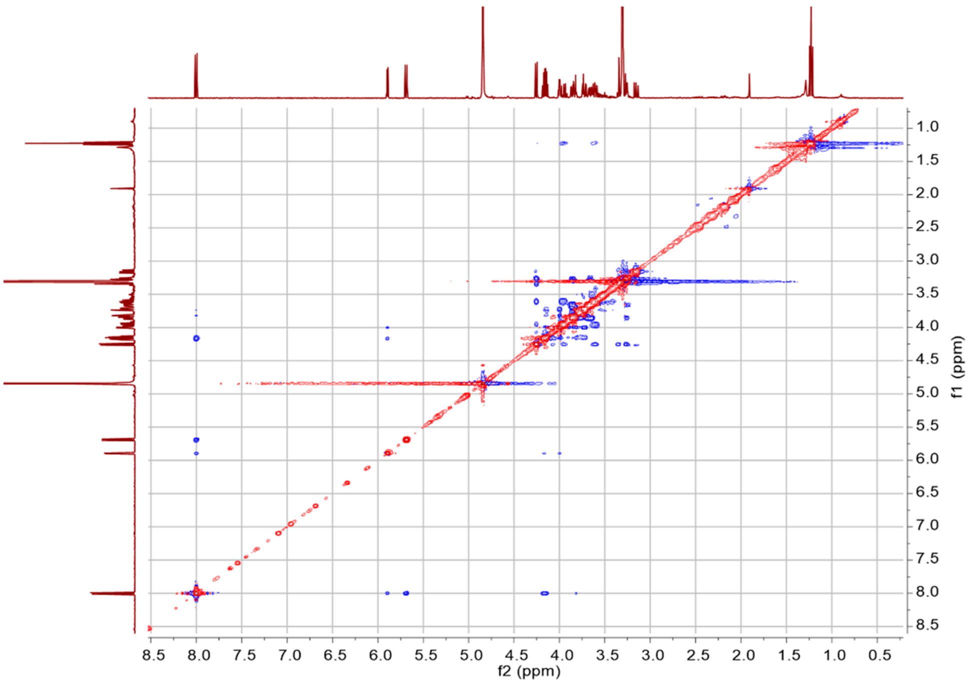

Figure S9. ^{1}H-^{1}H NOESY NMR spectrum of **1** (CD_3OD)

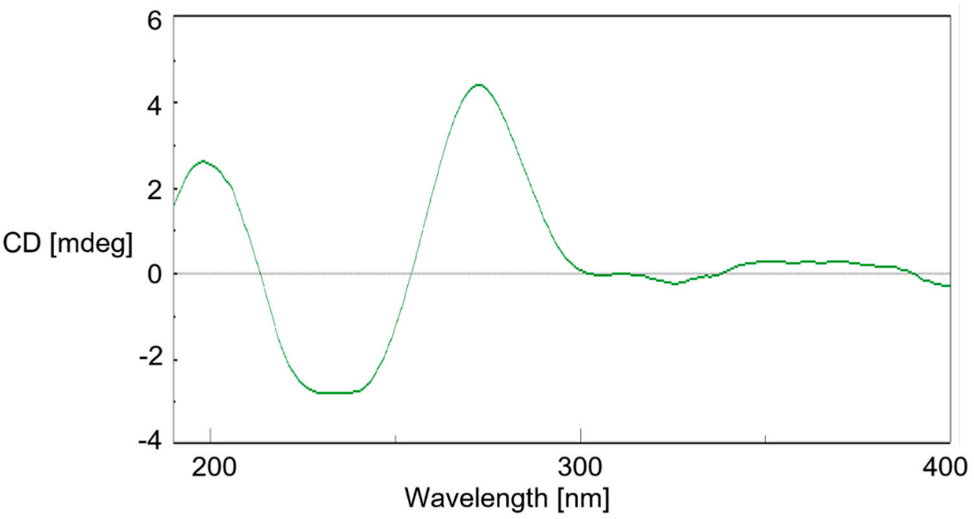

Figure S10. CD spectrum of **1**

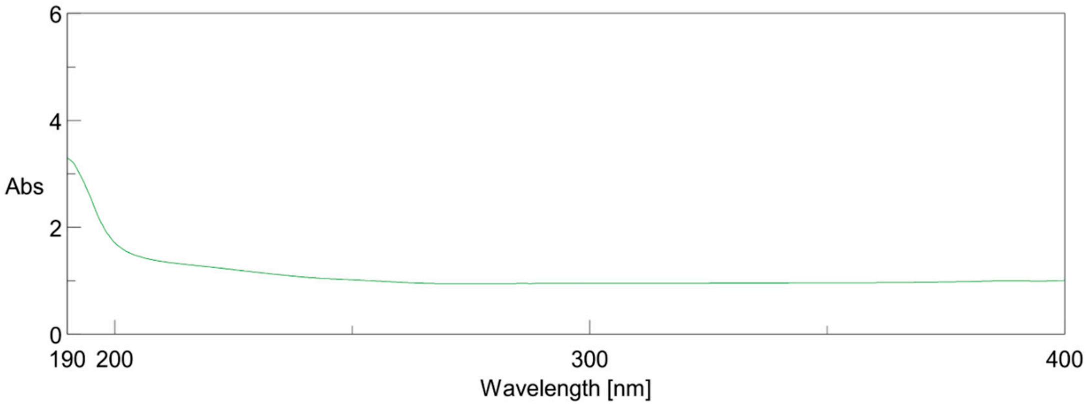

Figure S11. UV spectrum of **2**

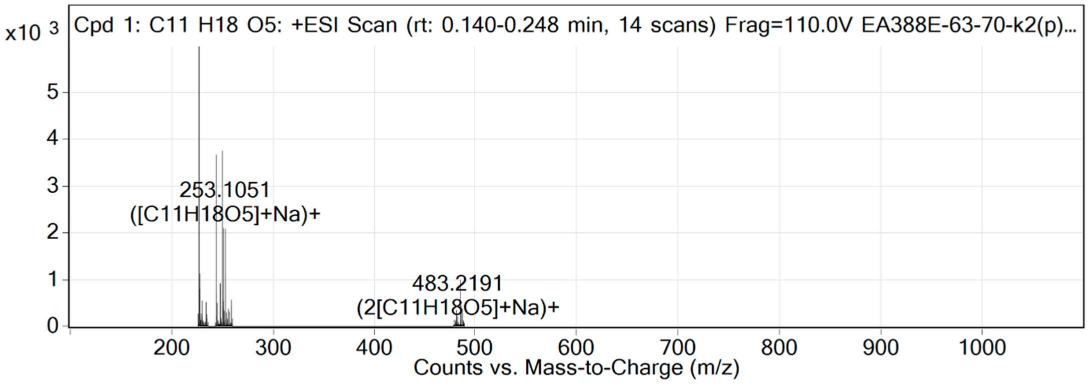

Figure S12. HRESIMS spectrum of **2**

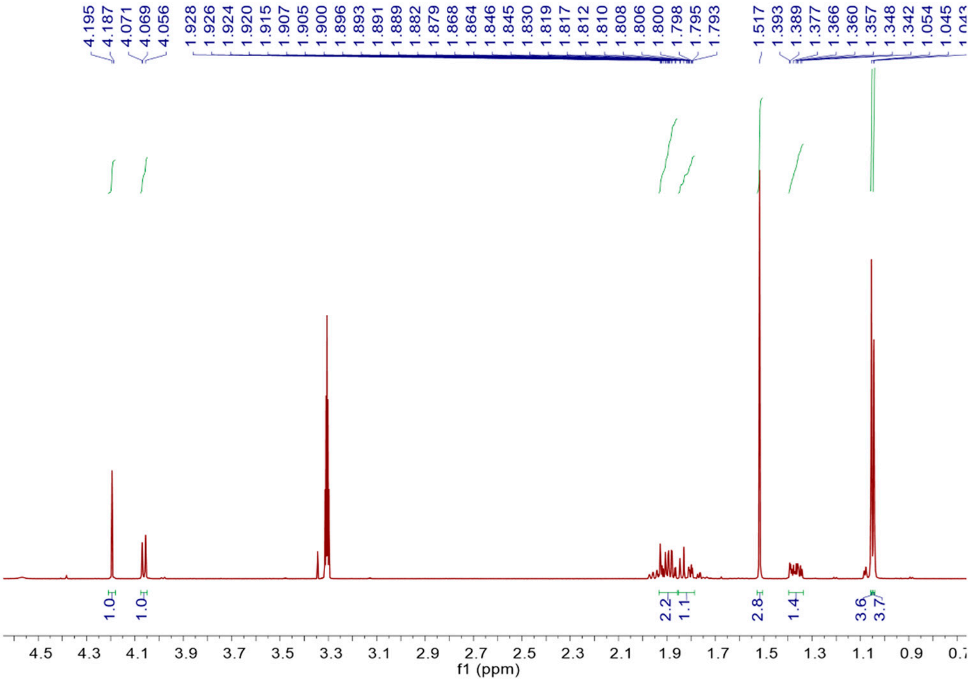

Figure S13. ^{1}H NMR spectrum of **2** (400 MHz, CD_3OD)

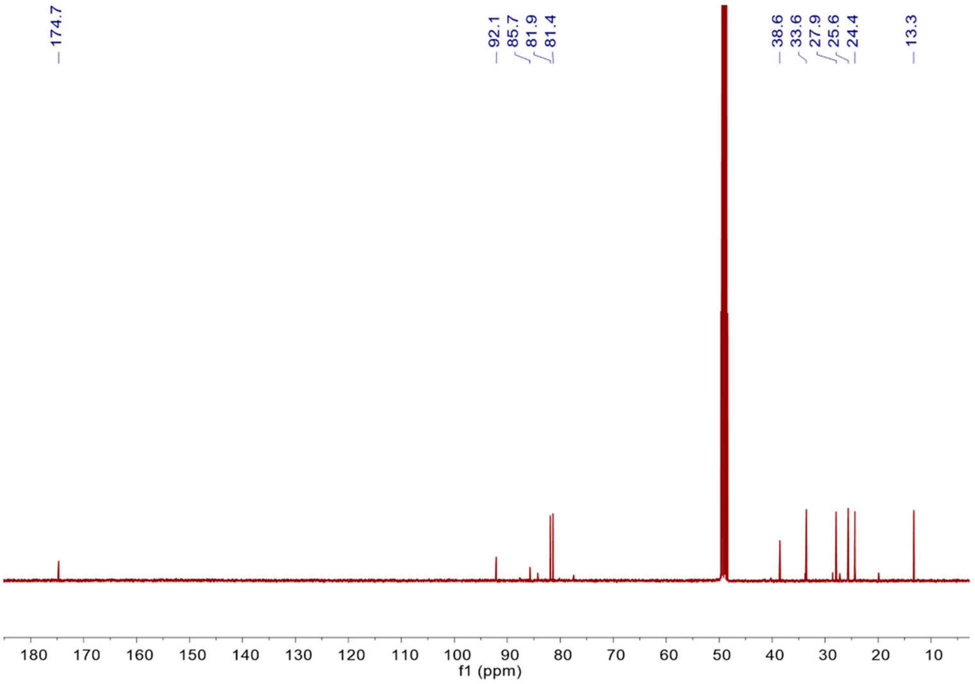

Figure S14. ^{13}C NMR spectrum of 2 (100 MHz, CD_3OD)

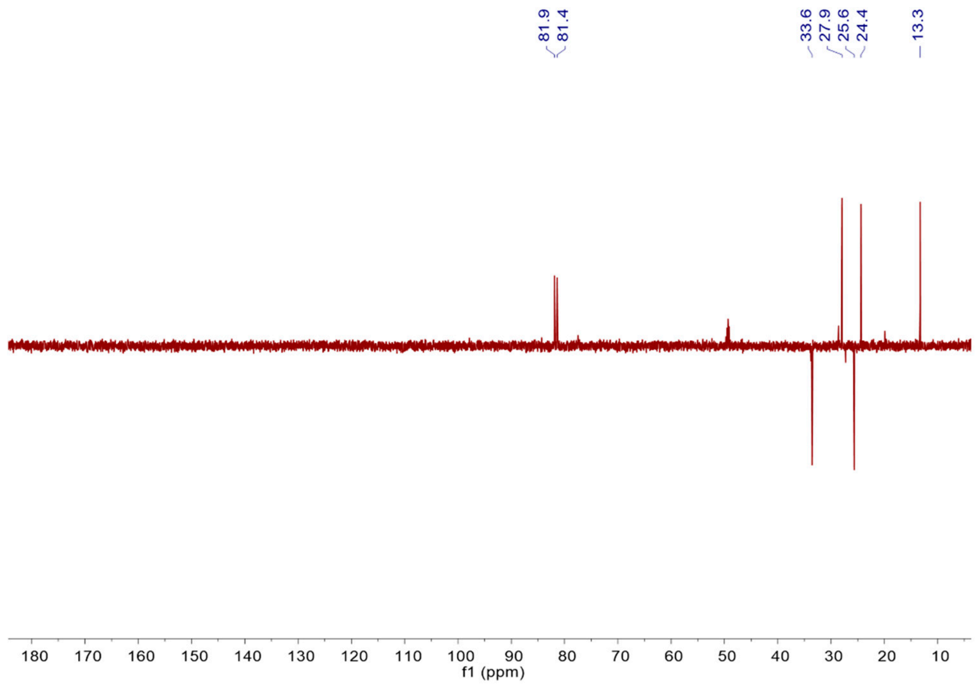

Figure S15. DEPT-135 NMR spectrum of **2** (100 MHz, CD3OD)

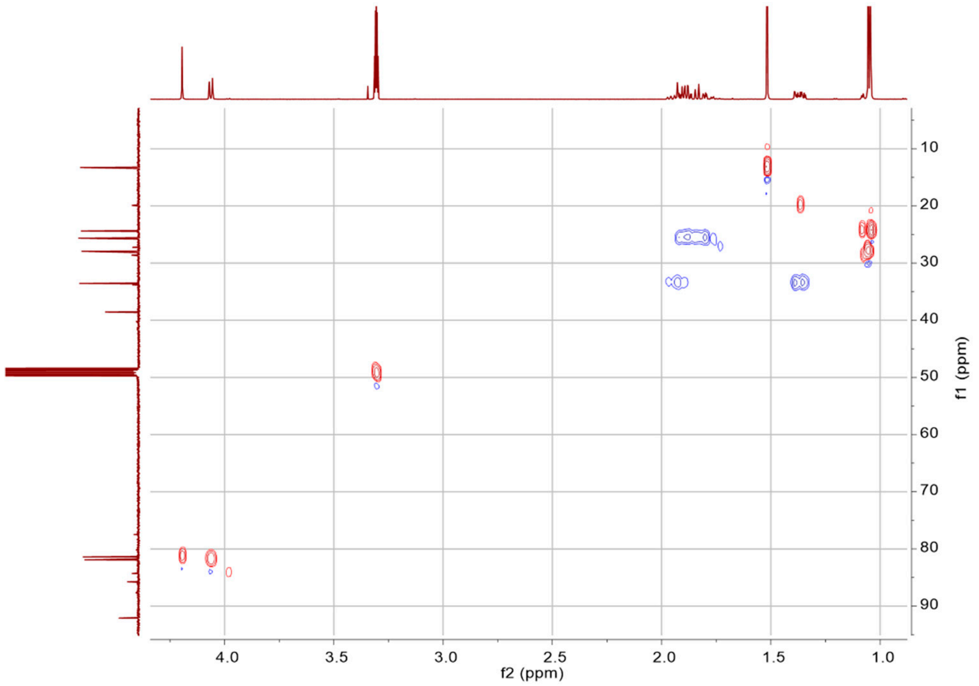

Figure S16. ^{1}H-^{13}C HSQC NMR spectrum of **2** (CD3OD)

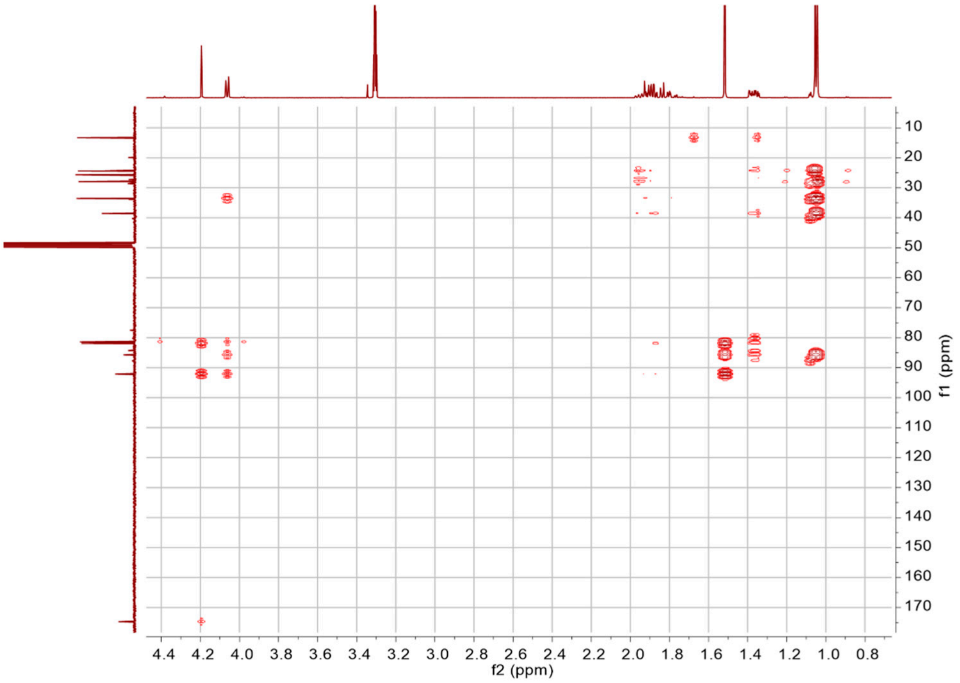

Figure S17. ^{1}H-^{13}C HMBC NMR spectrum of **2** (CD_3OD)

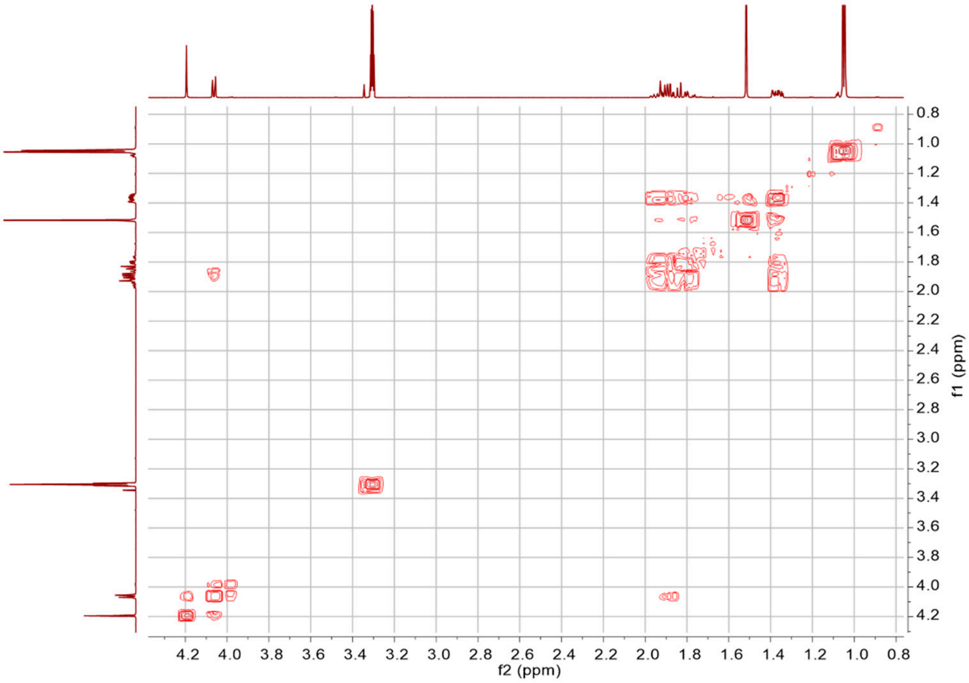

Figure S18. ^{1}H-^{1}H COSY NMR spectrum of **2** (CD_3OD)

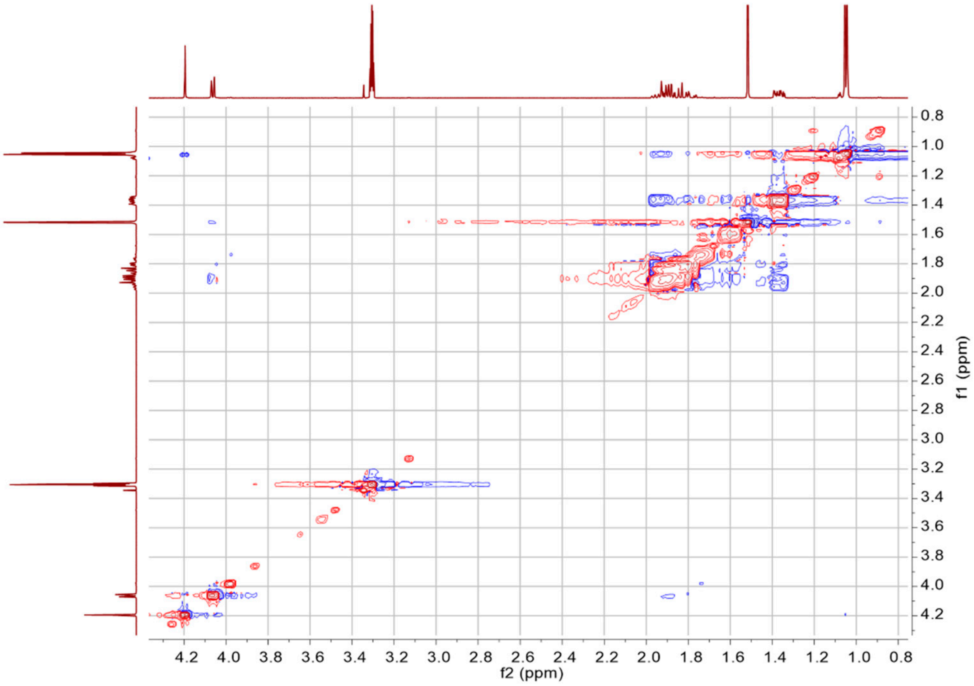

Figure S19. ^{1}H-^{1}H NOESY NMR spectrum of **2** (CD_3OD)

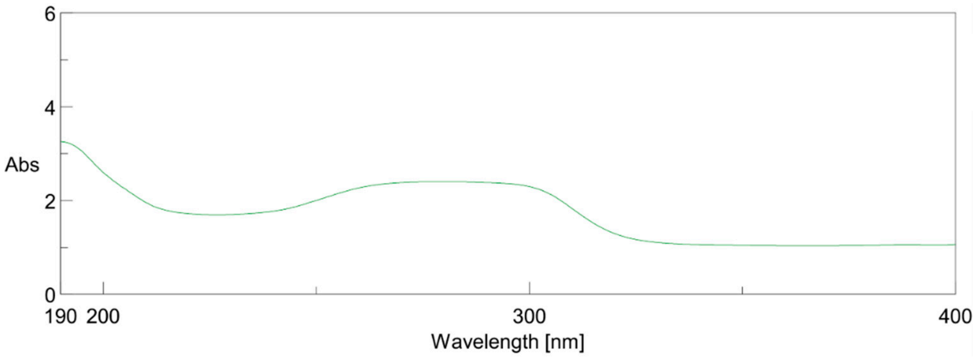

Figure S20. UV spectrum of **8**

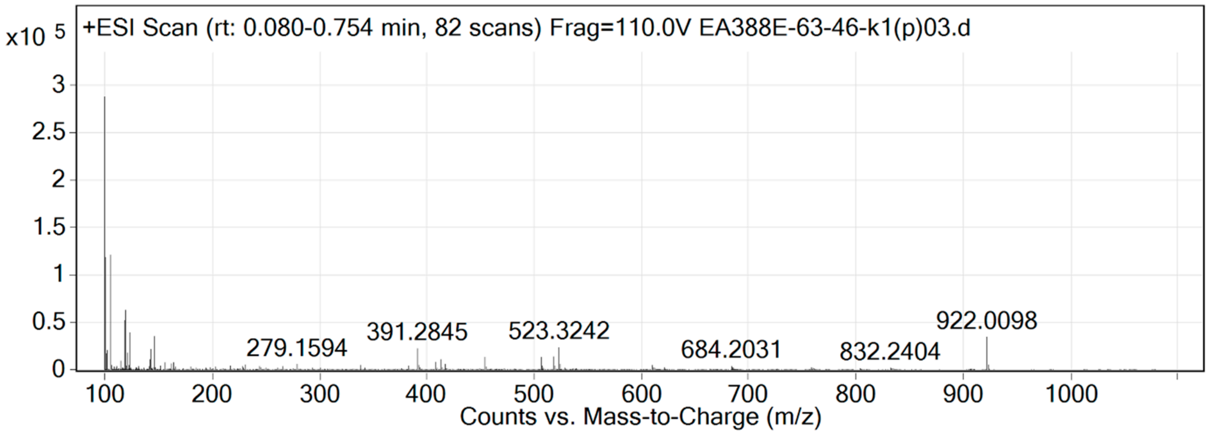

Figure S21. HRESIMS spectrum of **8**

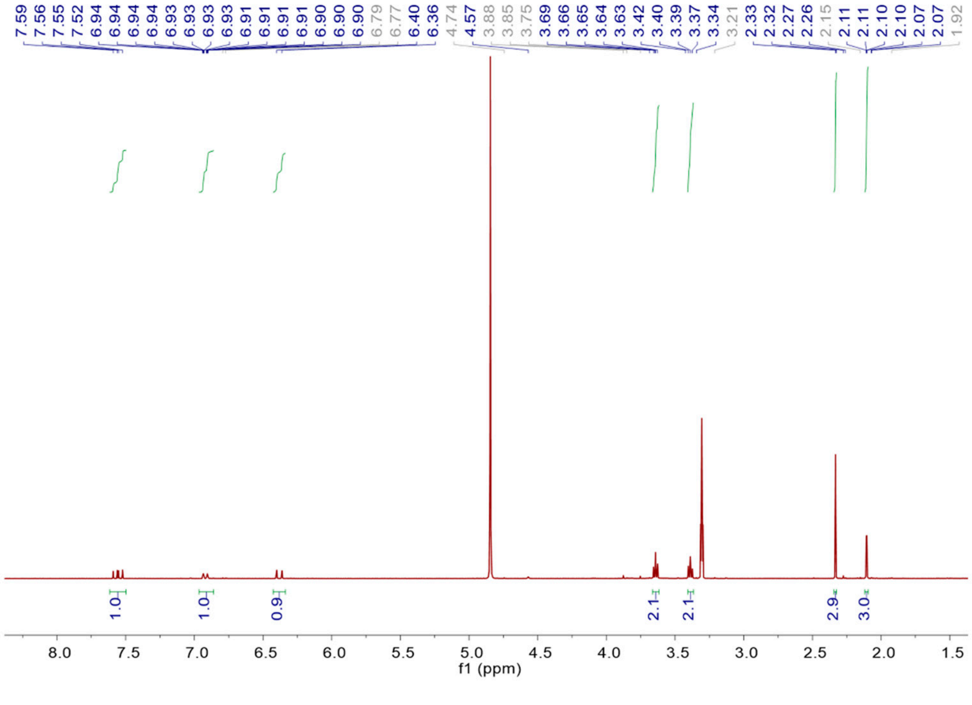

Figure S22. ^{1}H NMR spectrum of **8** (400 MHz, CD_3OD)

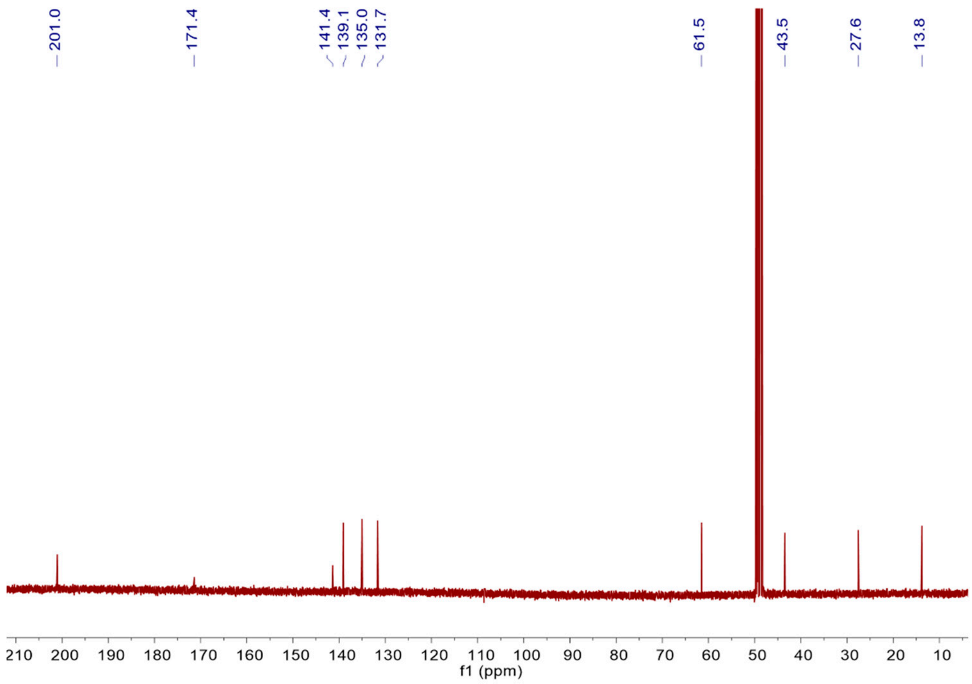

Figure S23. ^{13}C NMR spectrum of **8** (100 MHz, CD_3OD)

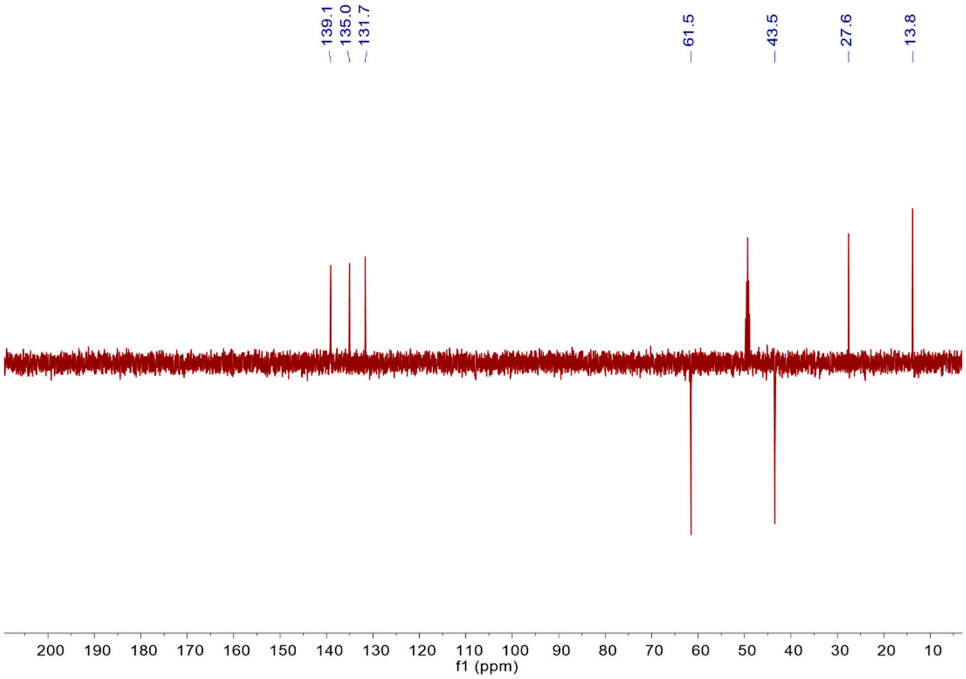

Figure S24. DEPT-135 NMR spectrum of **8** (100 MHz, CD_3OD)

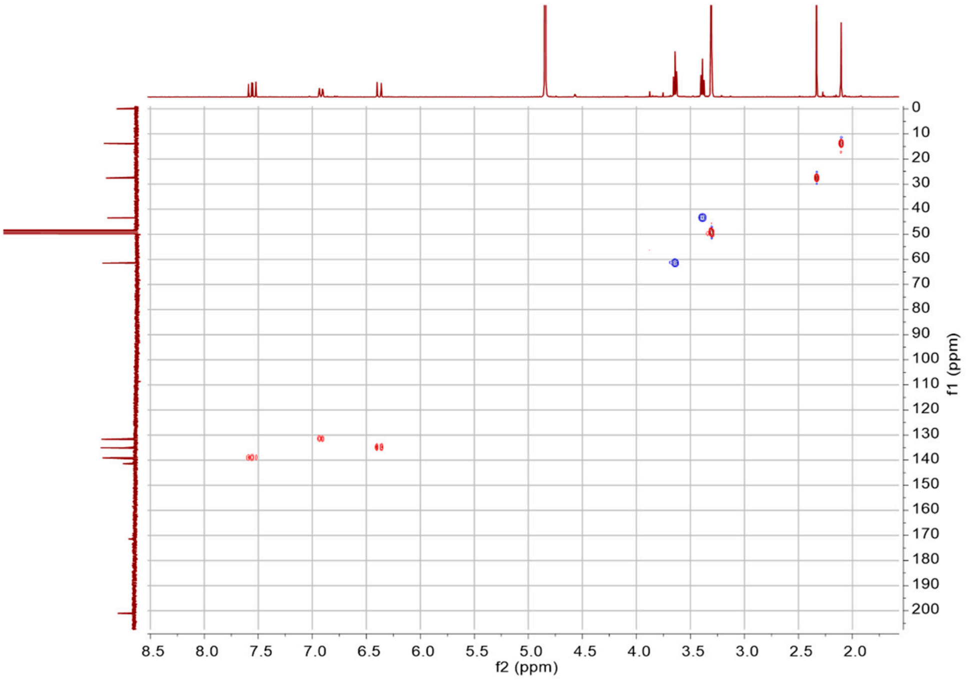

Figure S25. ^{1}H-^{13}C HSQC NMR spectrum of **8** (CD_3OD)

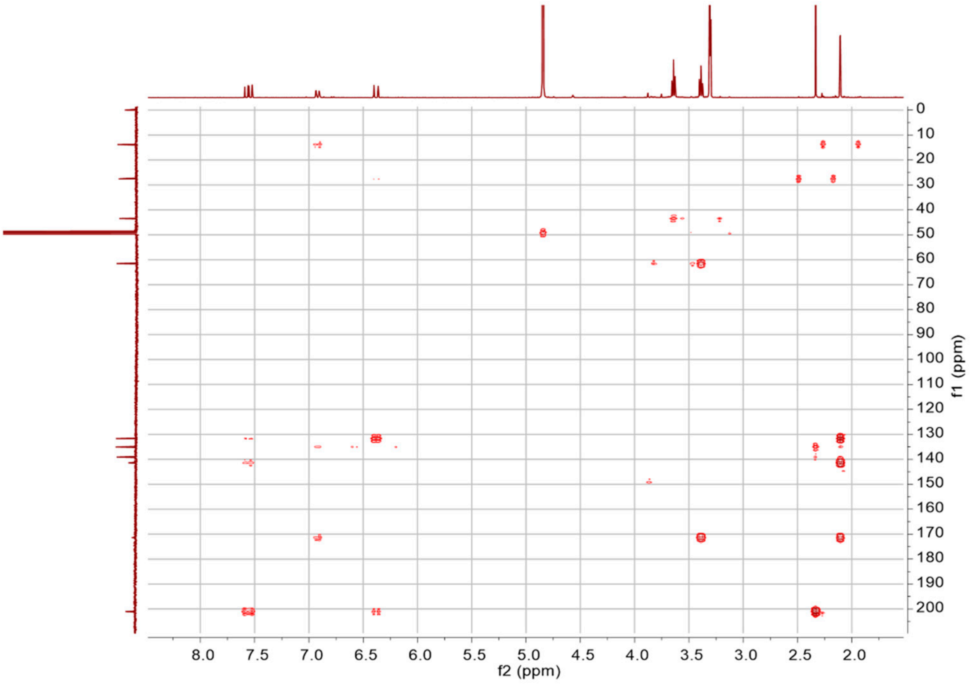

Figure S26. ^{1}H-^{13}C HMBC NMR spectrum of **8** (CD_3OD)

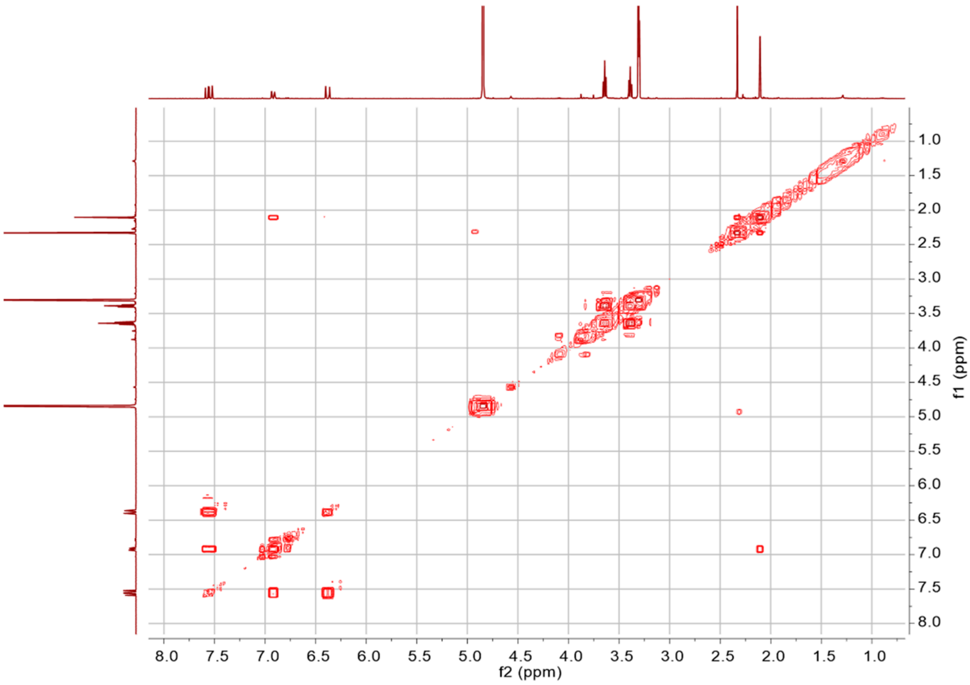

Figure S27. ^{1}H-^{1}H COSY NMR spectrum of **8** (CD_3OD)

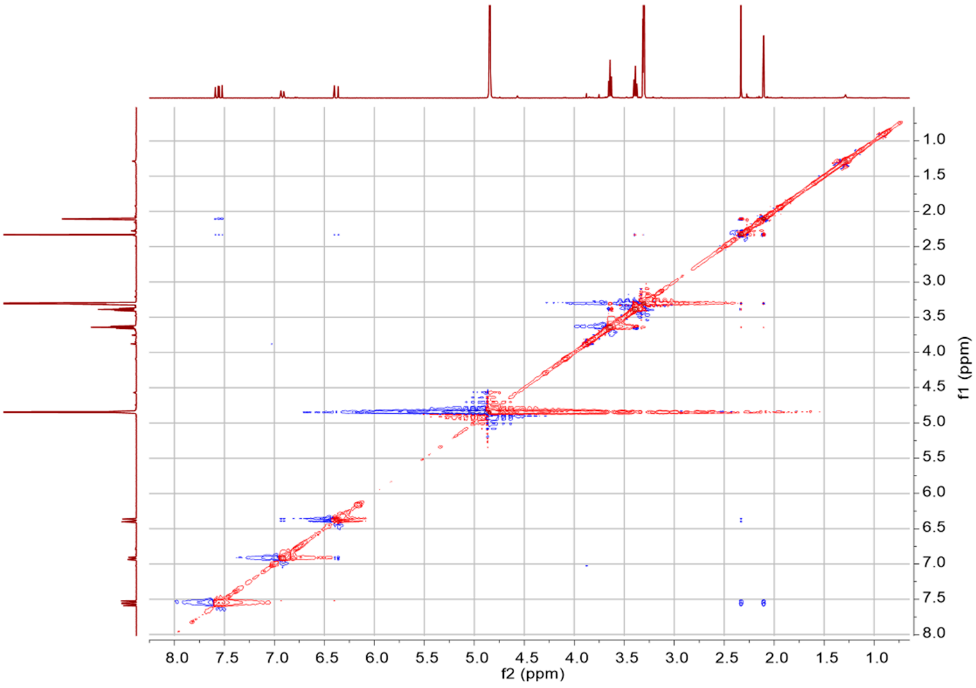

Figure S28. ^{1}H-^{1}H NOESY NMR spectrum of **8** (CD_3OD)

Table S1. *In silico* physicochemical and ADMET profiling of compound **1**.

Feature		ADMETlab2.0	SwissADME
**Physicochemical properties**	Van der waals (Volume)	434.2	434.4
	Density	387.7	
	Number of heavy atoms	1.1	
	Number of aromatic heavy atoms		30
	Number of hydrogen bond acceptors		6
	Number of hydrogen bond donors	13	11
	Number of rotatable bonds	6	6
	Number of rings	7	
	Number of atoms in the biggest ring	3	
	Number of heteroatoms	6	
	Formal charge	13	
	Number of rigid bonds	0	
	Flexibility	19	
	Stereo centers	0.4	
	TPSA (Topological polar surface area)	9	
	Molar refractivity	192.9	192.93
	Buffer LogS (log mol/L)		96.19
	Pure LogS (log mol/L)		
	LogP	-0.8	0.16
	LogD	-1.7	-2.79
	Buffer solubility (mg/L)	-1.8	
	Pure water solubility (mg/L)		
**Medicinal chemistry**	QED (Quantitative estimate of druglikeness)		
	SA score (Synthetic accessability score)	0.2	
	Fsp3	4.4	5.45
	MCE-18 (doi: 10.1021/acs.jmedchem.9b00004.)	0.8	0.76
	NP score (Natural product-likeness score).	75.6	
	Lipinski rule	1.6	
	Veber rule	Rejected	Rejected
	Pfizer rule		Rejected
	Egan rule	Accepted	
	GSK rule		Rejected
	Muegge rule	Rejected	
	Golden triangle		Rejected
	CMC-like rule	Accepted	
	Lead-like rule		
	MDDR-like rule		Rejected
	WDI-like rule		
	Ghose rule		

	PAINS (alert)		Rejected
	ALARM NMR (alert)	0	0
	BMS (alert)	0	
	Chelator rule (alert)	0	
	Brenk (alert)	0	
**Absorption**	Caco-2 cell permeability (log unit)		
	MDCK cell permeability (cm/s)	-6.4	
	Skin permeability (logKp, cm/hour)	1.1.E-04	
	Pgp-inhibitor probability		-11.63
	Pgp-substrate probability	2.E-03	
	Human intestinal absorption	0.6	No
	Bioavailability Score	1.0	Low
	20% Bioavailability probability		0.17
	30% Bioavailability probability	0.1	
**Distribution**	Plasma protein binding (%)		
	Volume distribution (L/kg)	13.8	
	BBB penetration probability	0.4	
	Fu (The fraction unbound in plasma %)	0.3	No
**Metabolism**	CYP1A2-inhibition probability		
	CYP1A2-substrate probability	0.003	No
	CYP2C19-inhibition probability	0.1	
	CYP2C19-substrate probability	0.03	No
	CYP2C9-inhibition probability	0.04	
	CYP2C9-substrate probability	0.001	No
	CYP2D6-inhibition probability	0.0	
	CYP2D6-substrate probability	0.0	No
	CYP3A4-inhibition probability	0.1	
	CYP3A4-substrate probability	0.004	No
**Excretion**	Clearance (mL/min/kg)		
	T1/2 (long half-life) probability	1.9	
**Toxicity**	hERG blockers probability		
	Human hepatotoxicity probability	0.04	
	Drug induced liver injury probability	0.4	
	Ames toxicity probability	1.0	
	Rat oral acute toxicity probability	0.2	
	FDAMDD (Maximum recommended daily dose) probability	0.002	
	Skin sensitization probability	0.002	
	Carcinogenicity probability	0.04	
	Eye corrosion probability	0.04	
	Eye irritation probability	0.003	

	Respiratory toxicity probability	0.01
	Acute algae toxicity	0.01
	Carcino_mouse	
	Carcino_rat	
	Acute daphnia toxicity	
	Acute medaka toxicity	
	Acute minnow toxicity	
	TA100_10RLI	
	TA100_NA	
	TA1535_10RLI	
	TA1535_NA	
**Environmental toxicity**	Bio-concentration Factors (-log10[(mg/L)/(1000*MW)])	
	IGC50 (-log10[(mg/L)/(1000*MW)])	0.4
	LC50 (-log10[(mg/L)/(1000*MW)])	1.0
	LC50DM (-log10[(mg/L)/(1000*MW)])	1.2
**Tox21 Pathway**	NR (Nuclear receptor)-AR probability	
	NR-AR-LBD probability	0.03
	NR-AhR probability	0.08
	NR-aromatase probability	0.002
	NR-ER probability	0.02
	NR-ER-LBD probability	0.5
	NR-PPAR-gamma probability	0.4
	SR-ARE probability	0.003
	SR-ATAD5 probability	0.2
	SR-HSE probability	0.03
	SR-MMP probability	0.003
	SR-p53 probability	0.01
**Toxicophore rules**	Acute toxicity rule (alerts)	
	Genotoxic carcinogenicity rule (alerts)	0
	Non-genotoxic carcinogenicity rule (alerts)	0
	Skin sensitization (alerts)	0
	Aquatic toxicity rule (alerts)	1
	Non-biodegradable rule (alerts)	0
	SureChEMBL rule (alerts)	1
